# Genome-wide transcriptome analysis of *Echinococcus multilocularis* larvae and germinative cell cultures reveals genes involved in parasite stem cell function

**DOI:** 10.3389/fcimb.2024.1335946

**Published:** 2024-01-25

**Authors:** Michaela Herz, Magdalena Zarowiecki, Leonie Wessels, Katharina Pätzel, Ruth Herrmann, Christiane Braun, Nancy Holroyd, Thomas Huckvale, Monika Bergmann, Markus Spiliotis, Uriel Koziol, Matthew Berriman, Klaus Brehm

**Affiliations:** ^1^ Consultant Laboratory for Echinococcosis, Institute of Hygiene and Microbiology, University of Würzburg, Würzburg, Germany; ^2^ Parasite Genomics, Wellcome Sanger Institute, Cambridge, United Kingdom; ^3^ Sección Biología Celular, Facultad de Ciencias, Universidad de la República, Montevideo, Uruguay

**Keywords:** echinococcosis, transcriptome, germinative cells, primary cells, differentiation, pluripotent, tumor necrosis factor, praziquantel (PZQ)

## Abstract

The lethal zoonosis alveolar echinococcosis is caused by tumour-like growth of the metacestode stage of the tapeworm *Echinococcus multilocularis* within host organs. We previously demonstrated that metacestode proliferation is exclusively driven by somatic stem cells (germinative cells), which are the only mitotically active parasite cells that give rise to all differentiated cell types. The *Echinococcus* gene repertoire required for germinative cell maintenance and differentiation has not been characterised so far. We herein carried out Illumina sequencing on cDNA from *Echinococcus* metacestode vesicles, from metacestode tissue depleted of germinative cells, and from *Echinococcus* primary cell cultures. We identified a set of ~1,180 genes associated with germinative cells, which contained numerous known stem cell markers alongside genes involved in replication, cell cycle regulation, mitosis, meiosis, epigenetic modification, and nucleotide metabolism. Interestingly, we also identified 44 stem cell associated transcription factors that are likely involved in regulating germinative cell differentiation and/or pluripotency. By *in situ* hybridization and pulse-chase experiments, we also found a new general *Echinococcus* stem cell marker, *EmCIP2Ah*, and we provide evidence implying the presence of a slow cycling stem cell sub-population expressing the extracellular matrix factor *Emkal1*. RNA-Seq analyses on primary cell cultures revealed that metacestode-derived *Echinococcus* stem cells display an expanded differentiation capability and do not only form differentiated cell types of the metacestode, but also cells expressing genes specific for protoscoleces, adult worms, and oncospheres, including an ortholog of the schistosome praziquantel target, EmTRPM_PZQ_. Finally, we show that primary cell cultures contain a cell population expressing an ortholog of the tumour necrosis factor α receptor family and that mammalian TNFα accelerates the development of metacestode vesicles from germinative cells. Taken together, our analyses provide a robust and comprehensive characterization of the *Echinococcus* germinative cell transcriptome, demonstrate expanded differentiation capability of metacestode derived stem cells, and underscore the potential of primary germinative cell cultures to investigate developmental processes of the parasite. These data are relevant for studies into the role of *Echinococcus* stem cells in parasite development and will facilitate the design of anti-parasitic drugs that specifically act on the parasite germinative cell compartment.

## Introduction

Alveolar echinococcosis (AE) is a lethal zoonosis prevalent in the Northern hemisphere, caused by larvae of the tapeworm *Echinococcus multilocularis* (Thompson, 2017). Infection of the intermediate host is initiated by oral uptake of infective eggs that are shed into the environment by definitive host faeces and contain the oncosphere stage. Once taken up orally by the intermediate host, the oncosphere hatches in the intestine, penetrates the intestinal epithelium, and gains access to the inner organs, of which the liver is primarily infected. Within the liver, the oncosphere undergoes a metamorphosis towards the metacestode, which are fluid-filled cysts surrounded by an acellular laminated layer, and an inner, cellular germinative layer. The germinal layer consists of a syncytial tegument with major functions in nutrient uptake from the host, as well as few differentiated cell types such as muscle and nerve cells (Brehm and Koziol, 2017; Thompson, 2017). About 25% of all cells within the GL are small, undifferentiated cells (so-called germinative cells; GC), for which we previously demonstrated stem cell properties (Koziol et al., 2014). We showed that GC are the only proliferative cell type in the metacestode, serve as a source for all differentiated cells of the GL, and are capable of self-renewal (Koziol et al., 2014). At later stages of the infection, initiated by GC, brood capsules are formed within the metacestode, which give rise to protoscoleces that are the infective form for the definitive host (Koziol et al., 2016a). Due to the almost unrestricted growth of the metacestode within the liver, AE leads to organ failure at later stages of the disease if not adequately treated. AE treatment is difficult and relies on anti-parasitic chemotherapy using benzimidazoles, which are, however, mostly parasitostatic and only rarely eliminate the parasite (Brunetti et al., 2010). It has thus been suggested that the high AE recurrence rates, which occur upon interruption of anti-parasitic chemotherapy (Brunetti et al., 2010), are due to limited activity of benzimidazoles against GC (Koziol and Brehm, 2015).

During recent years, several studies concerning the influence of host factors on GC dependent *Echinococcus* development have been carried out. Enhanced GC proliferation has, for example, been demonstrated in response to exogenously added mammalian insulin (Hemer et al., 2014), epidermal growth factor (EGF; Cheng et al., 2017b), fibroblast growth factor (FGF; Förster et al., 2019), or serotonin (Herz and Brehm, 2021). Very recently it has also been shown that the GC dependent differentiation of *E. multilocularis* towards the protoscolex is responsive to host and parasite cytokines of the transforming growth factor-β family (Kaethner et al., 2023). It is, however, unknown so far how these exogenous signals are integrated into gene regulatory networks within GC to affect differentiation of progeny cells. As in the case of the somatic stem cells of the related but free-living planarians, GC are morphologically undistinguishable but heterogenous regarding the expression of conserved regulators of pluripotency, indicating that sub-populations exist which differ in self-renewal and differentiation potential (Koziol et al., 2014). Several signalling components have been shown to be expressed in GC, but also in post-mitotic cells (Hemer et al., 2014; Förster et al., 2019; Kaethner et al., 2023). Furthermore, only few markers have been identified that are strongly associated with GC. Apart from GC markers deriving from mobile genetic elements (Koziol et al., 2015), the most important ones regarding stem cell regulation are the post-transcriptional regulators *em-nos-1* and *em-nos-2*, which are homologous to the general stem cell marker *nanos* (both are expressed in small sub-populations of GC; Koziol et al., 2014), as well as the transcriptional regulator EmSox2, which is related to human Sox2 (Cheng et al., 2017a). No data are presently available on GC gene regulatory networks governing maintenance of pluripotency, which is mostly due to a lack of information on stem cell-specific genes in *Echinococcus*. Likewise, although we previously introduced a primary cell cultivation system derived from stem cells from metacestode vesicles (Spiliotis et al., 2008; Spiliotis et al., 2010), which are strongly enriched in GC in early cultivation phases (Koziol et al., 2014), and can regenerate mature vesicles within 2 – 3 weeks (Spiliotis et al., 2010), there have been no in-depth analyses on GC sub-populations in this system, nor on the stem cell dynamics leading to the generation of mature vesicles. Hence, despite the importance of the *Echinococcus* GC system for parasite proliferation within the host, the mechanisms that govern GC maintenance and differentiation remain enigmatic.

Significant advances towards a characterization of flatworm stem cell subsets have recently been made by transcriptomic analyses, including single-cell sequencing, on the related organisms *Schmidtea mediterranea* and *Schistosoma mansoni* (Molinaro and Pearson, 2016; Fincher et al., 2018; Wendt et al., 2020; Li et al., 2021). Overall, these studies confirmed the presence of distinct stem cell subsets with differential potency and distinct differentiation fates (Fincher et al., 2018; Li et al., 2021). No comparable investigations have yet been carried out on stem cell systems of cestodes, except one study in which bulk transcriptomic analyses on X-ray treated adult worms of *Hymenolepis diminuta* identified genes potentially associated with cycling stem cells (Rozario et al., 2019). Other studies addressed the general protein encoding transcriptomic profiles of larvae and/or adult worms of *H. microstoma* (Olson et al., 2018; Preza et al., 2021), *Mesocestoides corti* (Basika et al., 2019), *Sparganum proliferum* (Kikuchi et al., 2021), *Dibothriocephalus dendriticus* (Sidorova et al., 2023), *Taenia pisiformis* (Zhang, 2019), and *T. multiceps* (Li et al., 2017). In *Echinococcus*, transcriptomic studies so far concentrated on *E. multilocularis* oncospheres (Huang et al., 2016), on different aspects of *E. granulosus* protoscoleces (Liu et al., 2017; Fan et al., 2020; Mohammadi et al., 2021; Yu et al., 2021; Pereira et al., 2022), or on the effects of electroporation on early *E. multilocularis* primary cell cultures (Pérez et al., 2022). However, none of these studies aimed at identifying the gene expression profiles of specific *Echinococcus* cell types.

We previously demonstrated that *Echinococcus* GC can be depleted from *in vitro* cultivated metacestode vesicles upon treatment with hydroxy urea (HU) or the Polo-like kinase inhibitor Bi-2536 (Koziol et al., 2014; Schubert et al., 2014). In the present work we utilized this methodology as a first step towards characterizing the stem cell associated transcriptome of metacestode GC. To this end, we compared the transcriptomes of control vesicles with HU or Bi-2536 treated vesicles and identified a core set of GC associated genes. We also carried out transcriptome analyses on *Echinococcus* primary cells, which are strongly enriched in GC. We validated our findings by performing RT-qPCR and *in situ* hybridization on selected genes and identified novel markers for *Echinococcus* GC and for GC sub-populations. Finally, by transcriptome sequencing and *in situ* hybridization, we also demonstrate that *Echinococcus* primary cells, which derived from *in vitro* cultivated metacestode vesicles, express not only metacestode factors, but also numerous genes specific to the protoscolex, oncosphere, and adult stages. The implications of these findings on future studies concerning *Echinococcus* stem cell function are discussed.

## Materials and methods

### Parasite material and *in vitro* cultivation

Experiments were carried out using the parasite isolates Ingrid, GH09, G8065, 7030, and MS1010, which derive from Old World Monkey species that had been naturally infected in a breeding enclosure (Tappe et al., 2007). Isolate H95 derived from a naturally infected fox of the Swabian mountains, Germany (Spiliotis et al., 2008). Metacestode tissue was propagated by intraperitoneal passage in Mongolian jirds (*Meriones unguiculatus*) essentially as previously described (Spiliotis and Brehm, 2009). At the time point of these experiments, all isolates except H95 were still capable of brood capsule and protoscolex production. Axenic metacestode vesicle cultivation was performed using conditioned medium from rat Reuber cells, nitrogen gas phase, and reducing conditions as previously described (Spiliotis and Brehm, 2009) with medium changes every 3 – 4 days. Axenically cultivated metacestode vesicles were treated with 40 mM HU or 150 nM Bi-2536 for 7 or 21 days, respectively, as previously described (Koziol et al., 2014; Schubert et al., 2014). After drug treatment, samples of the metacestode vesicles were recovered in conditioned medium for 24 h and then subjected to a 5 h EdU pulse to assess the reduction of stem cells capable of entering S-phase. Only vesicles in which EdU+ cells were reduced to less than 5% of the controls were further processed for transcriptomic analyses. Protoscoleces were isolated from *in vivo* cultivated parasite material as previously described (Koziol et al., 2013) and activated by low pH/pepsin/taurocholate treatment as described by Ritler et al. (2017). *E. multilocularis* primary cell cultures were set up essentially as previously described (Spiliotis et al., 2010) and were cultured for 2 days (PC1; small aggregates), 7 – 11 days (PC2; enlarged aggregates with cavities) or 16 – 22 days (PC3; onset of vesicle emergence) under axenic conditions. Samples of 2 days old primary cell cultures were tested by WISH specific for TRIM (Koziol et al., 2015) to assess GC enrichment. Only cultures with GC enrichment of over 70% were used for transcriptomic analyses. To measure effects of tumor necrosis factor α (TNFα) on parasite stem cells, primary cell cultures were set up as outlined above and 10 ng/ml (43 nM) recombinant human TNFα (Biomol, Hamburg, Germany) was added daily. The formation of mature metacestode vesicles was subsequently assessed as previously described (Förster et al., 2019).

### Nucleic acid isolation, cloning, and sequencing

Total RNA was isolated from *in vitro* cultivated metacestode vesicles using the RNEasy kit (Qiagen, Hilden, Germany) according to the manufacturer’s instructions and cDNA was generated using oligonucleotide CD3-RT essentially as previously described (Brehm et al., 2003). PCR products were cloned employing the TOPO TA cloning kit (Thermo Fisher Scientific) and sequenced by the Sanger method. In all cases, cDNA regions spanning the entire coding region plus 5’ and 3’ non-translated regions, as predicted according to transcriptome data, were PCR amplified using gene specific primers (listed in **Supplementary Table 1**). The corrected full-length sequences of all genes newly characterized in this study were submitted to the GenBank database and are available under the accession numbers listed in **Supplementary Table 1**.

### *In situ* hybridization, immunohistochemistry and EdU labelling

Whole-mount *in situ* hybridization (WISH) was performed on cultivated metacestode vesicles according to a previously established protocol (Koziol et al., 2014). Digoxygenin (DIG)-labeled probes were synthesized by *in vitro* transcription using the DIG RNA labelling kit (Roche) from cDNA fragment cloned into vector pJET1.2 (Thermo Fisher Scientific). Amplification primers for all probes used in this study are listed in **Table S1**. After hybridization, fluorescent specimens were processed and analyzed essentially as described recently (Koike et al., 2022; Kaethner et al., 2023). Control experiments using labeled sense probes were always negative. *In vitro* staining of S-phase stem cells was carried out as described (Koziol et al., 2014) using 50 µM 5-ethynyl-2’-deoxyuridine (EdU; Life Technologies, Darmstadt, Germany) for a 5 h pulse after vesicle isolation, followed by fluorescent detection with Alexa Fluor 555 azide as described previously (Koike et al., 2022; Kaethner et al., 2023). For *in situ* hybridization on primary cell preparations, the established protocol for metacestode vesicles (Koziol et al., 2016a) was slightly modified by including additional sedimentation steps during washing to avoid material loss. Gene expression patterns from all combined WISH/EdU labelling experiments were determined essentially as described (Koziol et al., 2016a) with vesicles from two biological replicates and 5 randomly chosen images of at least 5 different vesicles each. For all images, 100 cells were randomly marked in the DAPI (blue) channel and EDU+/WISH+ cells were then counted after addition of red/green channels. In the case of pulse-chase experiments, vesicles of three independently established cultures were used (biological triplicates). In each case, random pictures were taken from vesicles of three technical replicates and all nuclei (DAPI+) as well as all cells positive for EdU and *Emkal1* were counted from Z-stacks of 15 slices each. 3D images of these stacks were used to identify and eliminate false double positives. For statistical analysis, the ratios of *Emkal1*+ and EdU+ cells in relation to all cells of each single experiment were determined and mean values of the three replicates were then used for determining statistical differences by one-way ANOVA in GraphPad Prism 9.

### RT-qPCR

Total RNA was isolated from cultured vesicles as described above and cDNA was synthesized as described previously (Koziol et al., 2014). qPCR was then performed according to a previously established protocol (Pérez et al., 2019; Ancarola et al., 2020) on a StepOne Plus Realtime PCR cycler (Applied Biosystems). Primer sequences for all genes analyzed in this study are listed in **Table S1**. The constitutively expressed gene *elp* (EmuJ_000485800; Ancarola et al., 2020) was used as a control. Cycling conditions were 15 min at 95°C, followed by 40 cycles of 15 sec at 95°C, 20 sec of 58°C, and 20 sec of 72°C. PCR efficiencies were calculated using LinRegPCR (Untergasser et al., 2021), amplification product specificity was assessed by melting curve analysis and gel electrophoresis. Expression levels were calculated by the efficiency correction method using cycle threshold (Ct) values according to Ancarola et al. (2020).

### RNA-seq sample preparation

For identifying GC associated transcripts by RNA-Seq, metacestode vesicles (isolate: Ingrid) were cultivated under axenic conditions (Spiliotis and Brehm, 2009) and then treated for 7 days with 40 mM HU or for 21 days with 150 nM Bi-2536 (Axon Mechem, Groningen, Netherlands) as described previously (Koziol et al., 2014; Schubert et al., 2014). HU was added to the medium every day since it is unstable at 37°C and medium was changed every second day. In the case of Bi-2536, media changes were performed every second day with fresh drug administration. Bi-2536 control vesicles were incubated with comparable amounts of DMSO. After drug treatment, most vesicles were fixed for further analysis and small samples of unfixed vesicles were subjected to short term labelling (5 h pulse) with EdU as previously described (Koziol et al., 2014), to check for complete elimination of GC. Primary cells were isolated from *in vitro* cultivated metacestode vesicles of different isolates (**Supplementary Table 1**) and were set up in axenic culture essentially as previously described (Spiliotis et al., 2010). After 2 days (sample PC1), 7 - 11 days (PC2), and 16 - 22 days (PC3), cell aggregates were isolated and further processed for RNA isolation. All samples were washed three times with ice-cold PBS (phosphate buffered saline). *In vitro* cultivated metacestode vesicles were pierced with a needle prior to washing, to remove hydatid fluid. Samples were then transferred to TRIZOL ^®^-Reagent (Invitrogen, Karlsruhe, Germany) and stored at -80°C. Tissue samples were washed with ice-cold PBS before being mechanically homogenized in TRIZOL for 10 seconds. 200 µl of chloroform:isoamyl alcohol (24:1) was added and the samples were mixed thoroughly. Phase separation was carried out by centrifugation at 16,000 g at 4°C. 0.5X Isopropanol and 4 µl of glycogen (5mg/ml) were added to the aqueous phase, and the RNA was pelleted by centrifugation at 16,000 g at 4°C for 30 minutes. The resulting pellet was washed with 70% ethanol, air dried, and re-suspended in nuclease-free water. All samples of metacestode vesicles and primary cells were set up as three independently prepared biological replicates (n = 3).

### Library preparation and sequencing

Illumina transcriptome libraries were prepared using the Illumina TruSeq kit. Polyadenylated mRNA was purified from total RNA using oligo-dT dynabead selection followed by fragmentation by metal ion hydrolysis. First strand synthesis, primed using random oligonucleotides, was followed by 2nd strand synthesis with RNaseH and DNApolI to produce double-stranded cDNA. Template DNA fragments were end-repaired with T4 and Klenow DNA polymerases and blunt-ended with T4 polynucleotide kinase. A single 3’ adenosine was added to the repaired ends using Klenow exo- and dATP to reduce template concatemerization and adapter dimer formation, and to increase the efficiency of adapter ligation. Adapters containing primer sites for sequencing and index sequences were then ligated. Libraries made with the TruSeq protocol were amplified by PCR to enrich for properly ligated template strands, to generate enough DNA, and to add primers for flow-cell surface annealing, using Kapa HiFi enzyme. AMPure SPRI beads were used to purify amplified templates before pooling based on quantification using an Agilent Bioanalyser chip. Pooled TruSeq libraries were then pooled, and size selected (300 - 400bp fragments) using the Caliper. After adaptor ligation, individual libraries made with the Illumina mRNA-seq kit were size selected using the Caliper before PCR amplification followed by AMPure SPRI bead clean up and removal of adaptors with a second Caliper run. Kapa Illumina SYBR Fast qPCR kit was used to quantify the Illumina mRNA-seq libraries before pooling.

Libraries were denatured with 0.1 M sodium hydroxide and diluted to 6 or 8 pM in a hybridization buffer to allow the template strands to hybridize to adapters attached to the flow cell surface. Cluster amplification was performed on the Illumina cBOT using the V4 cluster generation kit following the manufacturer’s protocol and then a SYBR Green QC was performed to measure cluster density and determine whether to pass or fail the flow cell for sequencing, followed by linearization, blocking and hybridization of the R1 sequencing primer. The hybridized flow cells were loaded onto the Illumina sequencing platforms for sequencing-by-synthesis (100 cycles) using the V5 SBS sequencing kit then, *in situ*, the linearization, blocking and hybridization step was repeated to regenerate clusters, release the second strand for sequencing and to hybridize the R2 sequencing primer followed by another 100 cycles of sequencing to produce paired end reads. These steps were performed using proprietary reagents according to the manufacturer’s recommended protocol. Data was analysed from the Illumina GAIIx or HiSeq sequencing machines using the RTA1.8 analysis pipelines.

### Gene finding and annotation

For *E. multilocularis*, two genome versions have been published (Tsai et al., 2013). Gene models were predicted on version 3 (GCA_000469725.2), but genome statistics and additional analyses were conducted on an improved version 4 (GCA_000469725.3). To this end, gene models were transferred from genome version 3 to version 4 (10133 models transferred out of 10577). Since the new version of the genome is larger, new gene models were predicted that were not published previously, using Augustus v2.5.5 (Stanke et al., 2006). Annotations for new genes were assigned using BLAST searches against the NCBI nr database using custom scripts, and only unique gene models not overlapping with a previously identified model were retained. This resulted in a total of 10,699 gene models for genome version 4. The finished gene models were translated into protein predictions and domains were annotated using InterProScan v.5 (Jones et al., 2014). GO-terms were extracted from InterProScan results. Gene models and GO-terms are available through WormBaseParaSite WBPS7 (Howe et al., 2016).

### RNA-Seq mapping and calculation of expression levels

Mapping of sequencing reads was performed with Hisat2 v2.0.5 (Kim et al., 2015) using a maximum intron length of 40,000 base pairs. The number of uniquely mapped reads per transcript was calculated using HTSeqCount v0.7.1 (Anders et al., 2015) with a minimum quality score of 30. TPM values (Transcripts Per kilobase Million) were calculated for all transcripts.

### Differential expression calculation

Differential expression was calculated pairwise between datasets using DESeq2 v1.16.1 (Love et al., 2014) on statistical computation platform R v3.4.3 (Huber et al., 2015) with an adjusted p-value cut-off of 0.05. Adjustment for multiple testing was performed using the Benjamini-Hochberg procedure after independent filtering with genefilter v1.58.1 (Huber et al., 2015) (false discovery rate 0.05). To ascertain quality and correctness of differential expression analysis, fitting of the dispersion curve and outlier detection was assessed by plotting the dispersions (using plotDispEsts function) and the Cook´s distances for each comparison. For improved estimation of actual log2fold changes (FLC), function lfcShrink was used to calculate shrunken maximum a posteriori (MAP) LFCs. For quality control, both unshrunk maximum likelihood estimate (MLE) LFCs and MAP LFCs were visualized by plotting. Unless otherwise indicated, LFC in this work are MAP LFCs.

### GO enrichment analysis

GO enrichment analysis on GC associated genes was performed using topGO_2.28.0 (https://bioconductor.org/packages/release/bioc/html/topGO.html) on biological processed (BP) with “weight01” algorithm under the Fisher statistic. The gene universe consisted of all genes with a non-zero base mean.

### Bioinformatic procedures and statistical analyses

BLASTP searches were carried out against the *E. multilocularis* genome (version 5, January 2016; Tsai et al., 2013) on WormBase ParaSite (Howe et al., 2016) as well as against the SwissProt database as available at GenomeNet (https://www.genome.jp/). For BLASTP searches against *Schistosoma mansoni*, chromosomal assembly version 9 (GCA_000237925.5) was used (Protasio et al., 2012), in the case of *Schmidtea mediterranea*, assembly version ASM260089v1 (Rozanski et al., 2019), all as available under WormBase ParaSite (https://parasite.wormbase.org/index.html). Protein domain structure analysis was carried out using SMART 8.0 (http://smart.embl-heidelberg.de/). Multiple sequence alignments were performed using Clustal Omega (https://www.ebi.ac.uk/Tools/msa/clustalo/) and MEGA 11 software. Statistical analyses were performed using GraphPad Prism (version 9) employing one-way t-test or one-way ANOVA (as indicated).

## Results

### Identification of GC associated genes

As we previously showed, GC are specifically decimated in metacestode vesicles upon HU treatment without affecting differentiated cells (Koziol et al., 2014). We hypothesized that GC associated genes could be identified by comparing the transcriptomes of *in vitro* cultivated versus HU treated vesicles. To this end, metacestode vesicles were treated with 40 mM HU for 7 days and the reduction of EdU+ cells to less than 5% was measured by EdU incorporation assays. We then isolated RNA from treated and control vesicles, performed RNA-Seq, and mapped the resulting reads to the *E. multilocularis* genome (**Supplementary Table S2**). We identified 1,788 genes with statistically significantly (p ≤ 0,05) fewer mapped reads in the HU treated samples (**Supplementary Table S3**). To verify these data by an independent method, we also performed metacestode vesicle treatment with the Polo-like kinase inhibitor Bi-2536, for which we previously demonstrated that it specifically decimates *Echinococcus* GC in metacestode vesicles to less than 5% after 21 days treatment (Schubert et al., 2014). After treatment of *in vitro* cultivated vesicles with 150 nM Bi-2536 for 21 days, control of the reduction of EdU+ cells to less than 5%, RNA-Seq, and mapping, we identified 2,592 genes with significantly reduced expression in Bi-2536 treated vesicles compared to the DMSO control. Combination of these two datasets led to 1,184 genes that were significantly reduced in both HU and Bi-2536 treated vesicles (**Supplementary Table S3**). This set of genes should predominantly comprise factors that are specifically expressed in the GC compartment but may also contain genes that are expressed in GC and direct progeny, as well as genes expressed in differentiated cells, but dependent on the presence of GC. The 1,184 factors were termed GC associated genes (**Supplementary Table S3**).

We previously developed an *E. multilocularis* primary cell cultivation system in which cells isolated from metacestode vesicles are cultivated under axenic conditions in the presence of host cell conditioned medium (Spiliotis et al., 2008). This system yields completely regenerated metacestode vesicles within 2 – 3 weeks of incubation (Spiliotis et al., 2008; Spiliotis et al., 2010) and we also demonstrated strong enrichment for GC after 2 days of incubation (up to 80% versus ~21-25% in metacestode vesicles; Koziol et al., 2014; Koziol et al., 2015). We concluded that transcriptomic analyses on the primary cell cultivation system would aid us in verifying the set of genes associated with GC. We therefore performed RNA-Seq on *Echinococcus* primary cells after 2 days incubation and, again, mapped the resulting reads to the genome (dataset PC1). As can be deduced from **Supplementary Table S3**, 1,136 of 1,184 GC associated genes (96.0%) showed higher average read counts in PC1 when compared to metacestode vesicles from which these cultures derived, indicating that we had indeed identified a valid set of stem cell factors in *E. multilocularis*. Of these genes, 592 showed a statistically significant enrichment in PC1, whereas only 16 genes were statistically significantly reduced in PC1 when compared to control vesicles. Interestingly, among these genes we found *em-nos-2* (EmuJ_000606200) for which we previously showed specific expression in *E. multilocularis* stem cells in a pattern imlying an association with the developing nervous system (Koziol et al., 2014). This could be due to specific elimination of certain GC subsets in early developing primary cell cultures or to re-programming of GC under different culture conditions. We, finally, chose 10 well expressed factors among the set of GC associated genes and investigated their expression levels in HU treated and control vesicles by RT-qPCR in an independently performed cultivation experiment. As shown in **Supplementary Figure S1**, we verified the statistically significant reduction of expression of all 10 genes in HU treated vesicles.

Our RNA-Seq analyses indicated that the *E. multilocularis* metacestode expresses at least ~1,180 genes in a GC associated manner and that our dataset, verified by RNA-Seq on Bi-2536 treated vesicles and by RT-qPCR, provided a robust overview of these genes.

### Analysis of *E. multilocularis* GC associated genes

According to the function of GC in *Echinococcus* biology we expected that genes involved in proliferation, mitosis, and cell cycle control would be over-represented in the set of GC associated factors. To test this assumption, we performed comparative GO term analyses and, as shown in **Table 1**, genes associated with GO terms such as cell cycle checkpoint (GO:0000075), DNA replication initiation (GO:0006270), chromosome separation (GO:0051304), or cytokinesis (GO:0000910) were highly significantly enriched in the list of GC associated genes. Respective factors with particularly high differences in reads between HU treated and control vesicles encoded homologs to the cell cycle regulator kinase Wee1 (EmuJ_000659400), the mitotic serine/threonine kinase Aurora A (EmuJ_001059700), several origin recognition complex subunits (EmuJ_000764900; EmuJ_000046000; EmuJ_000045900; EmuJ_000880400; EmuJ_001056000) necessary for DNA replication, DNA primase (EmuJ_000487100), or the serine/threonine kinase MELK (maternal embryonic leucine zipper kinase; EmuJ_000500200), which in mammals is involved in cell cycle regulation and stem cell self-renewal (Nakano et al., 2005). Likewise, the list contains numerous genes encoding enzymes involved in nucleotide metabolism such as purine nucleoside phosphorylase (EmuJ_000635200), dUTP pyrophosphatase (EmuJ_000119100), or uridine phosphorylase (EmuJ_000734400). Interestingly, among the GC associated genes we also identified orthologs of *timeless* (EmuJ_000058900), which in *Drosophila* regulates circadian rhythm and in mammals most probably serves as a cell cycle checkpoint (Cai and Chiu, 2022), the tumor factor *p53* (EmuJ_00098700; Cheng et al., 2015; Wendt et al., 2022), and a member of the frizzled family of receptors for Wnt ligands (EmuJ_000636500) with conserved roles in the maintenance and expansion of stem cells (Clevers and Nusse, 2012).

**Table 1:** TOP 20 GO terms associated with *Echinococcus* germinal cell associated genes.

	GO.ID	Term	Annotated	Significant	Expected	weightFisher
1	GO:0006412	translation	242	63	29,93	1.6e-14
2	GO:0006260	DNA replication	69	27	8,53	1.3e-05
3	GO:0006281	DNA repair	75	26	9,28	9.0e-05
4	GO:0003333	amino acid transmembrane transport	10	6	1,24	0.00047
5	GO:0032508	DNA duplex unwinding	30	11	3,71	0.00056
6	GO:0007093	mitotic cell cycle checkpoint	5	4	0,62	0.00104
7	GO:0006270	DNA replication initiation	8	5	0,99	0.00116
8	GO:0035235	ionotropic glutamate receptor signaling pathway	13	6	1,61	0.00277
9	GO:0030071	regulation of mitotic metaphase/anaphase transition	7	4	0,87	0.00595
10	GO:0006298	mismatch repair	7	4	0,87	0.00595
11	GO:0000723	telomere maintenance	7	4	0,87	0.00595
12	GO:0009117	nucleotide metabolic process	115	17	14,22	0.00682
13	GO:0007156	homophilic cell adhesion via plasma membrane adhesion molecules	40	11	4,95	0.00744
14	GO:0022402	cell cycle process	41	17	5,07	0.01014
15	GO:0006505	GPI anchor metabolic process	17	3	2,1	0.01532
16	GO:0032269	negative regulation of cellular protein metabolic process	29	3	3,59	0.01540
17	GO:0000910	cytokinesis	5	3	0,62	0.01552
18	GO:0019219	regulation of nucleobase-containing compound metabolic process	297	34	36,73	0.01554
19	GO:2000112	regulation of cellular macromolecule biosynthetic process	309	35	38,22	0.01557
20	GO:0006165	nucleoside diphosphate phosphorylation	31	4	3,83	0.01570

Since we had previously conducted investigations into *Echinococcus* stem cell factors, we analysed whether these genes are present in the list of GC associated genes. We had already shown that the parasite genome contains two paralogs of the stem cell marker *nanos*, with *em-nos-1* (EmuJ_000861500) and *em-nos-2* (EmuJ_000606200) expressed in small sub-populations of EdU+ GC (Koziol et al., 2014), and, accordingly, both genes are present in the GC associated gene list. Although there are no true *piwi* orthologs in the *Echinococcus* genome (Tsai et al., 2013; Skinner et al., 2014), there are other genes encoding members of the Argonaute protein family which likely function in germline development and maintenance (Tsai et al., 2013). As we have previously shown, two of these genes, *em-ago-2A* (EmuJ_000739100) and *em-ago-2B* (EmuJ_000911700), are expressed in sub-populations of EdU+ GC of the germinal layer but are not confined to the stem cell compartment since they are also expressed in many post-mitotic cells (Koziol et al., 2014). This is reflected in our data. Although reads for both genes are slightly enriched in PC1 versus metacestode vesicles and reduced in HU- or Bi-2536 treated vesicles, the effects were not statistically significant. Hence, genes which are more ubiquitously expressed in both GC and post-mitotic cells are not contained in our list. Lastly, since we had previously shown that *Densovirus*-derived genes are deprived after HU treatment (Herz and Brehm, 2019), we also checked our list of GC associated genes for respective sequences and indeed found several gene copies for the non-capsid protein NS1 (EmuJ_000034800, EmuJ_00038860, EmuJ_002222800) and an associated gene for the capsid of the Pea enation mosaic virus (EmuJ_000034900, EmuJ_000388500) with drastically reduced read numbers in HU- and Bi-2536 treated vesicles when compared to the controls (**Supplementary Table S3**).

In addition to stem cell markers of the *nanos* family, we had previously shown by WISH and EdU incorporation experiments that several signalling factors are predominantly, though not exclusively, expressed in GC. This was the case for genes encoding the mitogen-activated protein kinase (MAPK) kinase kinase EmMEKK1 (EmuJ_000389600) and the MAPK EmMPK3 (EmuJ_000174000), which were expressed in approximately 60 – 70% of all metacestode GC (Stoll et al., 2021) and which are both contained in the list of GC associated genes (**Supplementary Table S3**). Likewise, we had demonstrated prominent GC expression of genes for the fibroblast growth factor receptor-like tyrosine kinase EmFR3 (EmuJ_000893600; Förster et al., 2019), the Wnt signalling factor Axin-2 (EmuJ_001141200; Montagne et al., 2019), the Polo-like kinase EmPlk1 (EmuJ_000471700; Schubert et al., 2014), and PIM kinase (Koike et al., 2022) and, again, all respective genes are among the GC associated factors identified by RNA-Seq (**Supplementary Table S3**). Similarly, both *Echinococcus* genes encoding Aurora kinases (EmuJ_001059700; EmuJ_00089100), previously shown to be expressed in GC by Cheng et al. (2019), are among the GC associated genes of our study. In contrast, numerous genes which we had identified to be predominantly expressed in muscle and nerve cells, encoding Wnt ligands (Koziol et al., 2016a) or TGFβ signalling components (Kaethner et al., 2023), are not part of the list.

Transcription factors are central components of stem cell regulatory networks that govern self-renewal and differentiation in metazoans (Molina and Cebrià, 2021). We therefore mined our list for transcription factor encoding genes that might play a role in *Echinococcus* stem cell function. Upon manual inspection of all genes of the list, supported by GO term analyses, we identified 44 transcription factors that are expressed in the metacestode in a GC associated manner (**Table 2**; **Supplementary Table S4**). In a previous study, Cheng et al. (2017a) had characterized an *Echinococcus* member of the Sox family of transcription factors, *EmSox2*, which could functionally replace murine *Sox2* during reprogramming of somatic cells to pluripotent stem cells, and which was expressed at the protein level in most proliferating GC. Notably, *EmSox2* (EmuJ_000704700) was also among the list of GC associated genes in our study with highly reduced read numbers in both HU- and Bi-2536 treated vesicles when compared to control vesicles (**Table 2**; **Supplementary Table S4**). Similar read count reductions upon HU and Bi-2536 treatment were observed for three genes encoding basic helix-loop-helix proteins (EmuJ_000098000; EmuJ_000451500; EmuJ_000627500), four zinc-finger transcription factors (EmuJ_000699600; EmuJ_000804100; EmuJ_000903400; EmuJ_001076200), two POU domain transcription factors (EmuJ_001026300; EmuJ_000449700), and another SOX family protein (EmuJ_000191100). Interestingly, the list of GC associated transcription factors also contained one member of the parasitic flatworm specific family of nuclear hormone receptors with two DNA-binding domains (Wu et al., 2007)(EmuJ_000240200), a member of the Gli-family of Krüppel-like factors (EmuJ_000711000), which are central components of the *hedgehog* signalling pathway (Petrov et al., 2017), the previously characterized, posteriorly expressed *hox* gene *Post2* (Koziol et al., 2016a; EmuJ_000959700), and orthologs to the transcription factor genes *FoxD* (EmuJ_000620400) and *tsh* (EmuJ_000580400), which are involved in body axis formation in planarians (Scimone et al., 2014; Vogg et al., 2014; Owen et al., 2015; Reuter et al., 2015). Notably, among the list of GC associated genes we also found an ortholog (EmuJ_001159300) to HDt_078513 from *H. diminuta*, which was found to be expressed in all stem cells of the neck region (Rozario et al., 2019), and which is also an ortholog of HmN_000137200 of *H. microstoma*, shown to be specific to the neck medullary parenchyma by Olson et al. (2018).

**Table 2:** Selection of *E. multilocularis* transcription factors associated with germinative cells.

Gene ID	annotation	TF class	PC1	HU	HU ctrl	Bi	Bi ctrl	Red HU	Red Bi
EmuJ_000098000	Bhlh factor math6	bHLH	16	0	6	0	4	100%	100%
EmuJ_000098700	Tumor protein p63	P53 family	150	7	49	0	32	86%	100%
EmuJ_000154900	Iroquois homeodomain protein IRX 6	homeobox	49	10	66	5	46	85%	89%
EmuJ_000191100	Transcription factor SOX 14	SOX family	66	3	27	3	32	89%	91%
EmuJ_000232900	Transcription factor SOX 6	SOX family	17	1	6	1	6	83%	83%
EmuJ_000240200	Nuclear receptor 2DBD gamma	Nuclear hormone receptor	9	1	3	1	4	66%	75%
EmuJ_000380300	LIM class homeodomain TF Lhx3	LIM/homeobox	7	1	3	0	4	66%	100%
EmuJ_000437800	Transcription factor SOX 14	SOX family	43	5	28	11	35	82%	69%
EmuJ_000451500	Basic helix loop helix dimer. region	bHLH	50	2	34	0	19	94%	100%
EmuJ_000580400	Protein tiptop	Zinc finger	31	3	13	7	22	77%	78%
EmuJ_000627500	Achaete scute transcription factor	bHLH	42	0	5	0	8	100%	100%
EmuJ_000638600	Zinc finger C2H2 type	Zinc finger	13	0	14	0	8	100%	100%
EmuJ_000699600	Zinc finger C2H2	Zinc finger	30	1	12	0	7	92%	100%
EmuJ_000704700	Transcription factor Sox1a	SOX family	2	0	1	0	1	100%	100%
EmuJ_000711000	Zinc finger transcription factor gli2	Zinc finger	8	0	2	1	3	100%	66%
EmuJ_000770300	ETS transcription factor Elf 2	ETS domain	57	4	43	1	45	91%	98%
EmuJ_000804100	Zinc finger protein	Zinc finger	35	0	18	0	12	100%	100%
EmuJ_000909600	MYB	Trihelix TF	63	2	25	0	24	92%	100%
EmuJ_000995700	Transcription factor 12	bHLH	41	3	17	3	13	82%	77%
EmuJ_001123200	Zinc finger protein	Zinc finger	109	6	14	2	15	57%	87%
EmuJ_001133050	Myeloid zinc finger 1	Zinc finger	65	15	29	17	35	48%	51%
EmuJ_001159300	Transcriptional factor nfil3:e4bp4	NFIL3	61	2	20	0	18	90%	100%
EmuJ_001163300	Zinc finger C2H2	Zinc finger	56	11	24	8	26	54%	69%
EmuJ_001177300	Basic leucine zipper TF	cAMP-dependent TF	64	2	11	5	23	82%	78%

Indicated are average TPMs in stem cell cultures (PC1) and in metacestode vesicles upon treatment with HU and Bi2536. Read reductions after treatment are listed. TF, transcription factor; bHLH, basic helix-loop-helix.

Taken together, our analyses of the list of GC associated genes revealed an enrichment for genes whose function would predict their expression in stem cells, all genes previously associated with GC, and numerous yet unstudied *Echinococcus* factors with presumed roles in stem cell maintenance and/or differentiation.

### Stem cell expression patterns of selected GC associated genes

To further verify GC expression of the genes on our list and to determine respective stem cell expression patterns, we performed WISH experiments combined with EdU-staining on *in vitro* cultivated metacestode vesicles. To this end, we chose eight genes with different expression levels in metacestode vesicles and with different degrees of read reduction upon HU and Bi-2536 treatment. All eight genes were full-length cloned and sequenced prior to WISH/EdU staining and are summarized in **Supplementary Figure S2**, including deduced amino acid sequences, homologies, and accession numbers.

**Figure 1:**
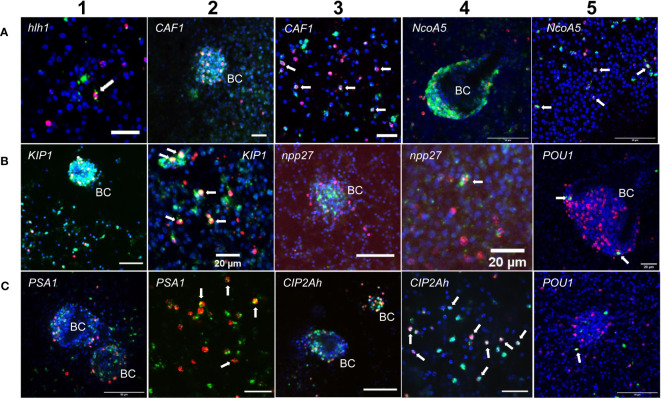
Expression of *E. multilocularis* genes in GC. WISH has been carried out for different *Echinococcus genes* (as indicated) on *in vitro* cultivated metacestode vesicles. Shown are merge pictures (single confocal slice) for three channels, in each case blue (DAPI, nuclei), red (EdU, S-phase GC), and green (WISH+). BC indicates developing brood capsules. White arrows mark cells double positive for EdU and WISH. Size bars represent 20 µm **(A-1, A-2, A-3, B-2, B-4, B-5, C-2, C-4)**, 40 μm **(B-1, B-3)**, and 50 μm **(A-4, A-5, C-1, C-3, C-5)**. For pictures showing separate channels, please refer to **Figures S2**.

Two of these genes, encoding a helix-loop-helix transcription factor (*Emhlh1*; EmuJ_000098000) and a POU domain protein (*EmPOU1*; EmuJ_000449700) displayed very low overall gene expression levels in the metacestode (≤ 6 TPM) but were reduced to zero reads upon HU treatment (**Table 3**; **Supplementary Table S3**). Accordingly, we only observed very few WISH+ signals for these genes but, in both cases, ~40% of all WISH+ cells were also EdU+ (**Table 3**; **Figure 1**). In the case of *EmPOU1*, we also identified EdU+/WISH+ cells in brood capsules (**Figure 1**), which generally display higher stem cell numbers and proliferative activity than the germinal layer (Koziol et al., 2014). Overall, ~8% and 2% of EdU+ cells also stained positive for *Emhlh1* and *EmPOU1*, respectively, indicating that both genes are expressed in small subsets of GC. Similarly, we found very low germinal layer cell numbers for a previously characterized gene encoding a neuropeptide (*Emnpp-27*; Koziol et al., 2016b) but, again, ~1% of all EdU+ cells also stained positive for *Emnpp-27* and 12% of all cells encoding the neuropeptide were positive for EdU (**Table 3**; **Figure 1**). Hence, although *Emnpp-27* codes for a neuropeptide with distinct expression pattern in the protoscolex nervous system (Koziol et al., 2016b), it is also expressed by a small subset of GC within the germinal layer.

**Table 3:** Expression of *E. multilocularis* germinative cell associated genes in S-phase stem cells.

Gene name	Gene ID	EdU+	WISH+	EdU+/WISH+	WISH+ of EdU+	EdU+ of WISH+
*EmCAF1*	EmuJ_000609400	7,7 ( ± 4,4)	19,8 ( ± 6,1)	4,0 ( ± 2,5)	52%	20%
*Emhlh1*	EmuJ_000098000	7,5 ( ± 1,6)	1,4 ( ± 0,7)	0,6 ( ± 0,3)	8%	43%
*EmKIP1*	EmuJ_001180200	11,6 ( ± 6,0)	19,4 ( ± 6,1)	6,7 ( ± 3,8)	58%	35%
*EmNcoA5*	EmuJ_001142000	8,5 ( ± 2,5)	13,6 ( ± 4,2)	4,3 ( ± 1,5)	50%	32%
*Emnpp27*	EmuJ_000347700	9,1 ( ± 3,5)	0,8 ( ± 0,4)	0,1 ( ± 0,05)	1%	12%
*EmPSA1*	EmuJ_000356700	8,4 ( ± 3,0)	13,8 ( ± 4,2)	3,9 ( ± 1,8)	46%	28%
*EmCIP2Ah*	EmuJ_000955000	7,9 ( ± 4,0)	21,4 ( ± 5,3)	7,8 (3,5)	99%	36%
*EmPOU1*	EmuJ_000449700	8,3 ( ± 3,6)	0,5 ( ± 0,3)	0,2 ( ± 0,1)	2%	40%

By combined in situ hybridization and EdU staining the number of S-phase stem cells (EdU+), WISH+ cells, and double positive cells (EdU+/WISH+) were determined in metacestode tissue. DWISH of EdU+ indicates the proportion of S-phase stem cells expressing the respective gene, DWISH+ of WISH+ indicates the proportion of gene expressing cells in S-phase. For each gene two independent cultures were investigated (N = 2) with 5 individual vesicles per culture and 5 randomly chosen images per vesicle (n = 25). Per image 100 cells were randomly chosen in DAPI channel (blue) and EdU+/WISH+ cells were subsequently determined in red/green channel (i.e. 5000 cells per WISH experiment in total). Standard deviation (in brackets) indicates variance per vesicle.

Two further genes, encoding homologs of puromycin-sensitive aminopeptidase (*EmPSA1*; EmuJ_000356700) and the nuclear receptor coactivator 5 (*EmNcoa5*; EmuJ_001142000), which has been implicated in stem cell function of planarians (Böser et al., 2013), were detected in ~14% of all germinal layer cells and in ~50% of all EdU+ cells (**Table 3**; **Figure 1**), indicating that they are expressed in substantial subsets of GC. Accordingly, we also found strong signals for both genes in developing brood capsules, which contain numerous proliferating stem cells (**Figure 1**). In planarians, the Ncoa5 encoding gene was expressed by nearly all stem cells (Böser et al., 2013). In *Echinococcus* this does not appear to be the case since expression in 14% of all germinal layer cells is clearly below the previously determined number of GC in this tissue (20-25%; Koziol et al., 2014; Koziol et al., 2015) and, at least according to our WISH/EdU analyses, 50% of S-phase stem cells did not express the gene (**Table 3**). We thus propose that *EmNcoa5* is expressed in a subset of GC. The expression of a puromycin-sensitive aminopeptidase (PSA) gene in a substantial fraction of *Echinococcus* stem cells requires further attention. Although PSA has so far mainly been studied in the context of neurodegeneration, it has been shown to associate with the spindle apparatus during mitosis of COS cells (Constam et al., 1995), which could be one of its functions in *Echinococcus*.

Two genes that were expected, based on homology, to be expressed in GC are *EmCAF1A* (EmuJ_000609400) and *EmKIP1* (EmuJ_001180200). *EmCAF1A* encodes a homolog of subunit A of chromatin assembly factor 1 (**Supplementary Figure S2**) that, in mammals, assembles histones onto replicating DNA during S-phase (Volk and Crispino, 2015). Upon HU treatment, read numbers of *EmCAF1A* were reduced to 28% (**Table 3**) and we found the gene expressed in ~50% of all EdU+ cells of the germinal layer (**Figure 1**, **Table 3**) as well as numerous *EmCAF1A*+ signals in developing brood capsules (**Figure 1**). In planarians, a homologous factor (p150) is expressed in many neoblasts (although not exclusively) and RNAi knockdown of the respective gene resulted in severe regeneration defects and neoblast depletion (Zeng et al., 2013), indicating a role in stem cell maintenance. We thus consider a role of *EmCAF1A* in *Echinococcus* GC maintenance and epigenetic control highly likely. *EmKIP1* encodes a kinesin-like protein with highest similarity to human KIF11 (also known as Eg5) that is required for establishing a bipolar spindle during mitosis (Wordeman, 2010). In our analyses, we found 58% of EdU+ cells were also positive for *EmKIP1* (**Table 3**, **Figure 1**), supporting an important function of the gene in GC. Accordingly, we also detected intense *EmKIP1* signals in developing brood capsules (**Figure 1**). Given the important role of human KIF11 in mitosis (Wordeman, 2010), we initially expected that all GC express *EmKIP1*. However, since the protein is specifically needed during mitosis, it is possible that its expression is initiated towards the end of S-phase, thus explaining why only ~60% of GC were found *EmKIP1*+. Furthermore, the *E. multilocularis* genome also encodes a second kinesin-like protein with homology to the *EmKIP1* gene product (EmuJ_000198400), which is also among the list of GC associated genes (**Supplementary Table S3**) and which could, at least in part, functionally replace *EmKIP1*.

**Figure 2:**
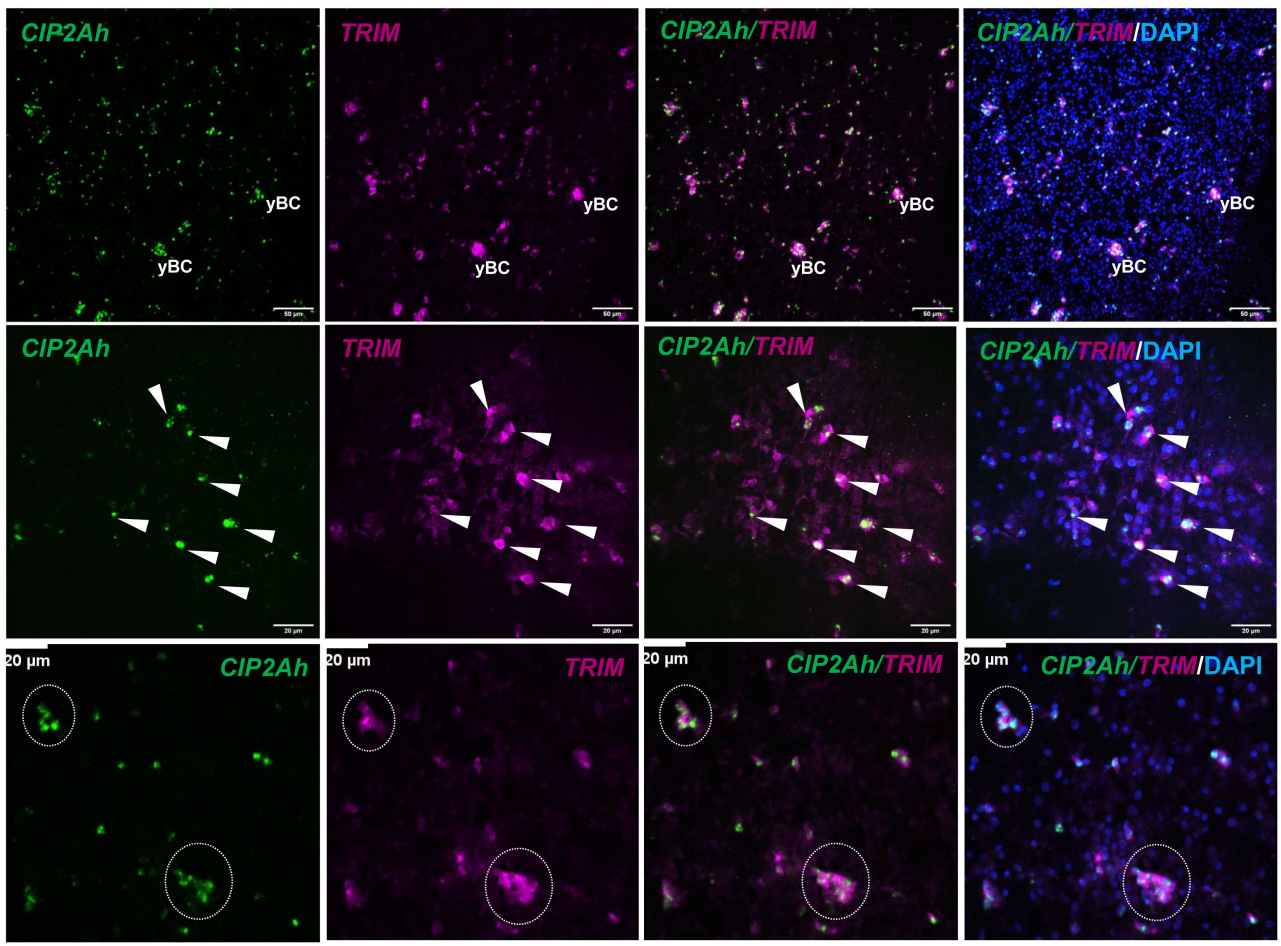
*EmCIP2Ah* as a general marker of *E. multilocularis* GC. Double WISH has been carried out on *in vitro* cultivated metacestode vesicles using probes specific for *EmCIP2Ah* and *EmTRIM*. Upper panel: Overview of metacestode tissue showing from left to right, *EmCIP2Ah*+ cells (green), *EmTRIM*+ cells (magenta), overlay of *EmCIP2Ah* and *EmTRIM*, and merge of all channels (including DAPI, blue). yBC indicates young brood capsules. Size bar represents 50 μm. Middle panel: Region of metacestode with dispersed GC. White triangles indicate cells double positive for *EmCIP2Ah* and *EmTRIM*. Size bar represents 20 μm. Note the diffuse, cytoplasmic signal for *EmTRIM* and the focused, nuclear signal for *EmCIP2Ah*. Lower panel: Metacestode tissue with accumulations of GC (circles). Note that individual GC cannot be distinguished by *EmTRIM*+ signal, but by *EmCIP2Ah*+ signal. Size bar represents 20 μm. All images shown are single confocal slices.

Finally, we investigated *EmCIP2Ah* (EmuJ_000955000) that was expressed in ~21% of all cells of the germinal layer (**Table 3**; **Figure 1**). As shown in **Supplementary Figure S2**, *EmCIP2Ah* encodes a protein with no discernible functional domains, but which is distantly related to human cellular inhibitor of PP2A (CIP2A), an inhibitor of tumour suppressor activities of protein phosphatase 2A in human cancer, e. g. by de-phosphorylating the transcription factor c-Myc (Denk et al., 2021). Interestingly, in our combined EdU/WISH analyses, we found intense *EmCIP2Ah* signals in developing brood capsules and, within the germinal layer, almost 100% of all EdU+ cells were also positive for *EmCIP2Ah* (**Figure 1**, **Table 3**). Together with the total number of *EmCIP2Ah*+ cells in the germinal layer, which agrees with the total number of GC in this tissue (21 – 25%; Koziol et al., 2014; Koziol et al., 2015), we assumed that *EmCIP2Ah* could be a general marker of *Echinococcus* GC, irrespective of the cell cycle. To clarify the situation, we carried out double WISH with *EmCIP2Ah* and *TRIM* (terminal-repeat retrotransposon in miniature), the so far only known general stem cell marker of *Echinococcus* (Koziol et al., 2015). As shown in **Figure 2**, we found almost 100% congruence between the signals for both genes, indicating that *EmCIP2Ah* indeed is a marker of all GC in the germinal layer. Whether *EmCIP2Ah* fulfils similar roles in *Echinococcus* as CIP2A in humans remains to be established. Although *Echinococcus* encodes a close homolog of PP2A (EmuJ_001106500), c-Myc is absent in cestodes (Tsai et al., 2013), thus excluding a similar mechanism in the parasite. Conversely, it has recently been shown that mammalian CIP2A also complexes with TOPBP1 (DNA topoisomerase 2 binding protein 1) in a mitosis-specific genome maintenance complex (De Marco Zompit et al., 2022). Since a close homolog of TOPBP1 is also expressed by *Echinococcus* (EmuJ_000898700), interactions between these two factors could contribute to the maintenance of genomic stability during GC mitosis.

Taken together, our combined EdU/WISH analyses verified stem cell expression of factors listed as GC associated genes and even identified one new general *Echinococcus* stem cell marker (*EmCIP2Ah*), which will facilitate future investigations on *Echinococcus* stem cell dynamics.

### *Emkal1* may mark a slow cycling GC subpopulation

Although morphologically indistinguishable, flatworm stem cell populations are heterogenous on gene expression level and in both planaria and schistosomes, functionally different sub-populations have been described (reviewed in Molina and Cebrià, 2021; You et al., 2021). Typically, these sub-populations can be distinguished by preferential expression of sub-type marker genes such as the transcription factor *soxP-1* in the case of planarian σ-neoblasts (Molina and Cebrià, 2021) or the nuclear hormone receptor *eled* in schistosome ϵ-cells (Wang et al., 2018). Although we previously noted clear differences in gene expression profiles between GC and neoblasts (Koziol et al., 2014; Förster et al., 2019), we were interested whether at least some of the known flatworm maker genes can be used to distinguish different classes of GC. We identified an *E. multilocularis* ortholog (EmuJ_000888900) to *S. mediterranea kal-1*, which is present in the list of GC associated genes (**Supplementary Table S3**). *S. mediterranea kal-1* encodes a homolog of the mammalian extracellular matrix glycoprotein Anosmin-1 and is highly specifically expressed in a ζ neoblast sub-population, as well as in ventrally located, early epidermal progenitors that derive from ζ neoblasts (Wurtzel et al., 2017). We therefore performed experiments concerning the expression of *Emkal1* in GC and in GC progeny.

**Figure 3:**
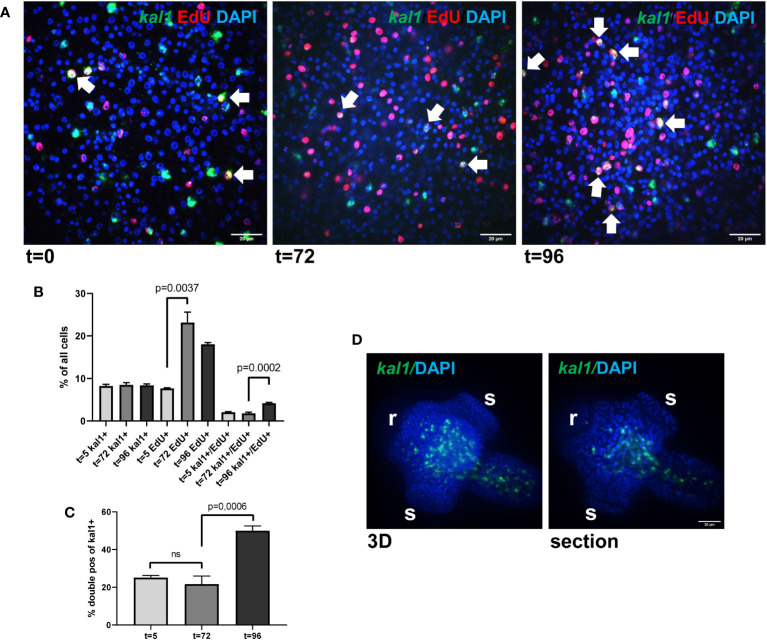
*Emkal1* as a potential marker for a slow cycling GC subpopulation. **(A)** WISH/EdU staining for metacestode vesicles at different time points as indicated. Displayed are merge pictures (single confocal slices) of three channels, blue (DAPI, nuclei), red (EdU, S-phase GC), and green (WISH, *Emkal1*). White arrows indicate cells double positive for EdU and *Emkal1*. Size bar repesents 20 μm. **(B)** Counts of cells positive for *Emkal1* (*kal1*+), positive for EdU (EdU+), and double positive (*kal1*+/EdU+) of all cells in metacestode vesicles at different time points (as indicated). Displayed are mean values ± standard deviation. Each experiment has been carried out three times independently (N = 3) with technical triplicates of which in each case 3 images of 3 individual vesicles were analysed. **(C)** Percentage of double positive *Emkal1*+/EdU+ cells among all *Emkal1*+ cells at different time points (as indicated), deduced from values displayed in **(B)**. Displayed are mean values ± standard deviation. Statistical analysis was carried out using one-way ANOVA and statistically significant differences are indicated by bars with respective p-values. The total numbers of metacestode cells counted (n) for each condition were 8,176 (t=0), 8,652 (t=72), and 10,058 (t=96). **(D)** WISH for *Emkal1* on protoscoleces. Shown are a 3D project (20 sections) of one protoscolex (3D) and one section of the same protoscolex (section). Size bar represents 20 μm.

We isolated metacestode vesicles from culture, subjected them to a 5 h pulse of EdU incorporation, fixed part of the vesicles, and performed WISH specific for *Emkal1*. The remaining vesicles were then cultured for another 3 (72 h) or 4 days (96 h) prior to fixation and *Emkal1* specific WISH. By independent WISH analyses we had previously shown that approximately 8% of all metacestode cells stain positive for *Emkal1* and, accordingly, at t=0 we found 8.2% (± 0.4%) of metacestode vesicle cells (n = 8,176) positive for *Emkal1* (**Figure 3**). As expected, the relative proportion of *Emkal1*+ cells among all metacestode cells did not change over the incubation period of 4 days (**Figure 3**). We then analysed the proportion of cells that stained positive for both *Emkal1* and EdU. At t=0 we found 7.7% (± 0.1%) of all cells (n = 8,652) positive for EdU, which increased to 23.2% (± 2.5%) and 18.0% (± 0.4%) after 3 and 4 days (n = 10,058) of incubation, respectively (**Figure 3**). Although we cannot exclude that a certain proportion of the increase in EdU+ cells resulted from a delayed incorporation of EdU that was stored within vesicle fluid after the 5 h pulse (Koziol et al., 2014), the statistically significant increase in EdU+ cells after three days indicated that a large proportion of GC that had incorporated EdU at t=0 had undergone mitosis at this time point, resulting in two EdU+ progeny cells. Interestingly, the initial number of cells which stained positive for both *Emkal1* and EdU (2.1 ± 0.1% of cells) had not changed after 3 days (1.8 ± 0.2%) of further culture. However, after 4 days of incubation, this compartment had a statistically significant, approximately 2-fold increase to 4.2 (± 0,2%) of all cells. Likewise, the proportion of *Emkal1*+/EdU+ cells among *Emkal1*+ cells showed no statistical difference between t=0 and t=3d (25.1 ± 1.1% versus 21.6 ± 4.3%) but significantly increased to 49.9 ± 2.5% at t = 4d (**Figure 3**), indicating that *Emkal1* might be expressed in a GC sub-population with a prolonged cell cycle. The sharp increase of EdU+/*Emkal1*+ cells would then either be explained by asymmetric cell division of an EdU+ GC, resulting in another EdU+ GC and an EdU+ differentiating cell, or by delayed incorporation of EdU by a GC subpopulation with a cell cycle that is 24 h longer, compared to the bulk of metacestode GC.

In *Schmidtea*, *kal1* serves as a marker for ventral identities and most *kal1*+ cells are located close to the ventral epidermis, where they partially co-localize with the epidermis marker *DCLK2* (Wurtzel et al., 2017). To investigate whether *Emkal1* could have a similar function, we performed WISH on protoscoleces but as shown in **Figure 3**, we exclusively obtained *Emkal1* signals in central areas of the neck and regions around the anterior and posterior ring commissures of the nervous system. Based on these findings, we suggest that *Emkal1*+ GC and progeny cells are not directly involved in the formation of Echinococcus surface structures.

Taken together, our analyses verified that *Emkal1*, which is listed among our GC associated genes, is expressed in a significant subpopulation (25%) of *Echinococcus* GC and might serve as a marker for stem cells with a prolonged cell cycle. In contrast to the function of planarian *kal1*, however, we did not obtain evidence for an involvement of *Emkal1* in tegument formation. Future experiments, including double WISH for *Emkal1* and markers for terminally differentiated *Echinococcus* cells, will be required for elucidating the precise role of *Emkal1*+ GC in *Echinococcus* development.

### GC in *Echinococcus* primary cell cultures show an expanded differentiation capacity

**Figure 4:**
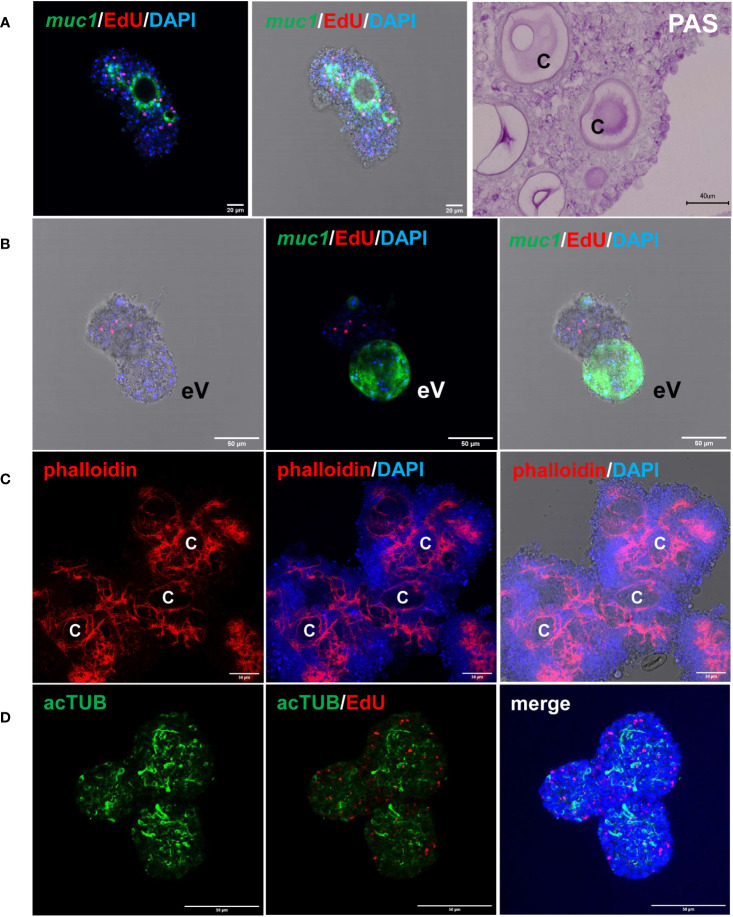
Features of *E. multilocularis* primary cell culture aggregates. **(A)** Expression of laminated layer components. *muc1* indicates WISH for *muc1* on 7 d old aggregate. Displayed are (left) merge of three channels (blue, DAPI, nuclei; red, EdU, S-phase GC; green, WISH, *muc1*) and (middle) merge combined with brightfield. Size bar represents 20 μm. Note intense *muc1* signal at layer surrounding cavity. PAS indicates PAS staining of 10 d old aggregate section. Size bar represents 40 μm. **(B)** *muc1*-specific WISH on 14 d old aggregate with emerging vesicle (eV). Shown are bright field with EdU and DAPI (left), merge of EdU, DAPI, and WISH (middle), and merge of bright field and all three channells (right). Size bar represents 50 μm. **(C)** Phalloidin staining of 10 d old aggregates, marking muscle fibres. Displayed are from left to right: phalloidin staining (red, muscle), phalloidin plus DAPI (blue, nuclei), and phalloidin plus DAPI plus bright field. C indicates cavity. Size bar represents 50 μm. **(D)** Nerve cell staining of 10 d old aggregate using a-AcTub antibody. Displayed are from left to right: α-AcTub (green, nerve cells), α-AcTub plus EdU (red, S-phase GC), merge of α-AcTub, EdU, and DAPI (blue, nuclei). Size bar represents 50 μm. All images show single confocal slices.

We previously established an *E. multilocularis* primary cell cultivation system in which parasite cells, isolated from cultivated metacestode vesicles, are seeded into wells and are further cultivated under axenic conditions (Spiliotis et al., 2008; Spiliotis et al., 2010). After 2 days of incubation, the primary cells regularly form small aggregates that are highly enriched with GC (up to ~80%), but also contain some differentiated cell types such as muscle or nerve cells (Koziol et al., 2014). Upon further incubation, the primary cell cultures develop larger aggregates with internal cavities and, after 2 – 3 weeks, reveal fully developed metacestode vesicles (Spiliotis et al., 2008; Spiliotis et al., 2010). We further characterized the development of primary cell aggregates into metacestode vesicles and found increasing numbers of muscle and nerve cells within the aggregates after 7 days of culture (**Figure 4**), which apparently derived from differentiation of stem cells into definitive cell fates. Expression of *Em-muc-1* (EmuJ_000742900; Koziol et al., 2014), a member of *Echinococcus*-specific family of apomucins that are major components of the LL (Díaz et al., 2011), is considered a hallmark of a functional metacestode tegument. We therefore carried out *Em-muc-1* specific WISH on primary cell aggregates at different time points of development and found strong *Em-muc-1*+ signals surrounding early and late cavities (**Figure 4**), indicating that these first emerge as outside-in structures. Using PAS staining to mark LL components, we identified masses of PAS positive structures on sections of primary cell aggregates (**Figure 4**), which had obviously been secreted into the lumen of the parasite primary cell cavities. At later stages of primary cell development, we identified emerging vesicles displaying intense *Em-muc-1*+ signals close to the surface, which were still connected with aggregates, but showed the regular morphology of mature metacestode vesicles, with a distal laminated layer, secreted by the parasite tegument. We thus assume that the formation of mature metacestode vesicles by primary cell cultures starts with the formation of *Em-muc-1*+ tegumental cells that surround internal cavities in an outside-in arrangement and secrete laminated layer material into the cavities. Once these internal tegumental structures gain contact to the surface of the aggregate, either by apoptosis or by further differentiation of cells between cavity and aggregate surface, they then invert into regularly shaped metacestode vesicles that eventually emerge from the main bodies of the aggregates.

To further characterize differentiation mechanisms within primary cell aggregates, we complemented our Illumina transcriptome data obtained after 2 days of cultivation (PC1) by analyses on aggregates at later stages of development. We chose cultures in which the primary cell aggregates were already enlarged and contained internal cavities, but which did not yet show emerging vesicles (PC2; after 7 - 11 days) and cultures in which vesicles were about to emerge (PC3; after 16 – 22 days). As with metacestode vesicles after HU and Bi-2536 treatment, the resulting reads were mapped to the genome (**Supplementary Table S2**). For a selection of genes, we also carried out combined EdU/WISH experiments on primary cell cultures at later time points of development.

**Figure 5:**
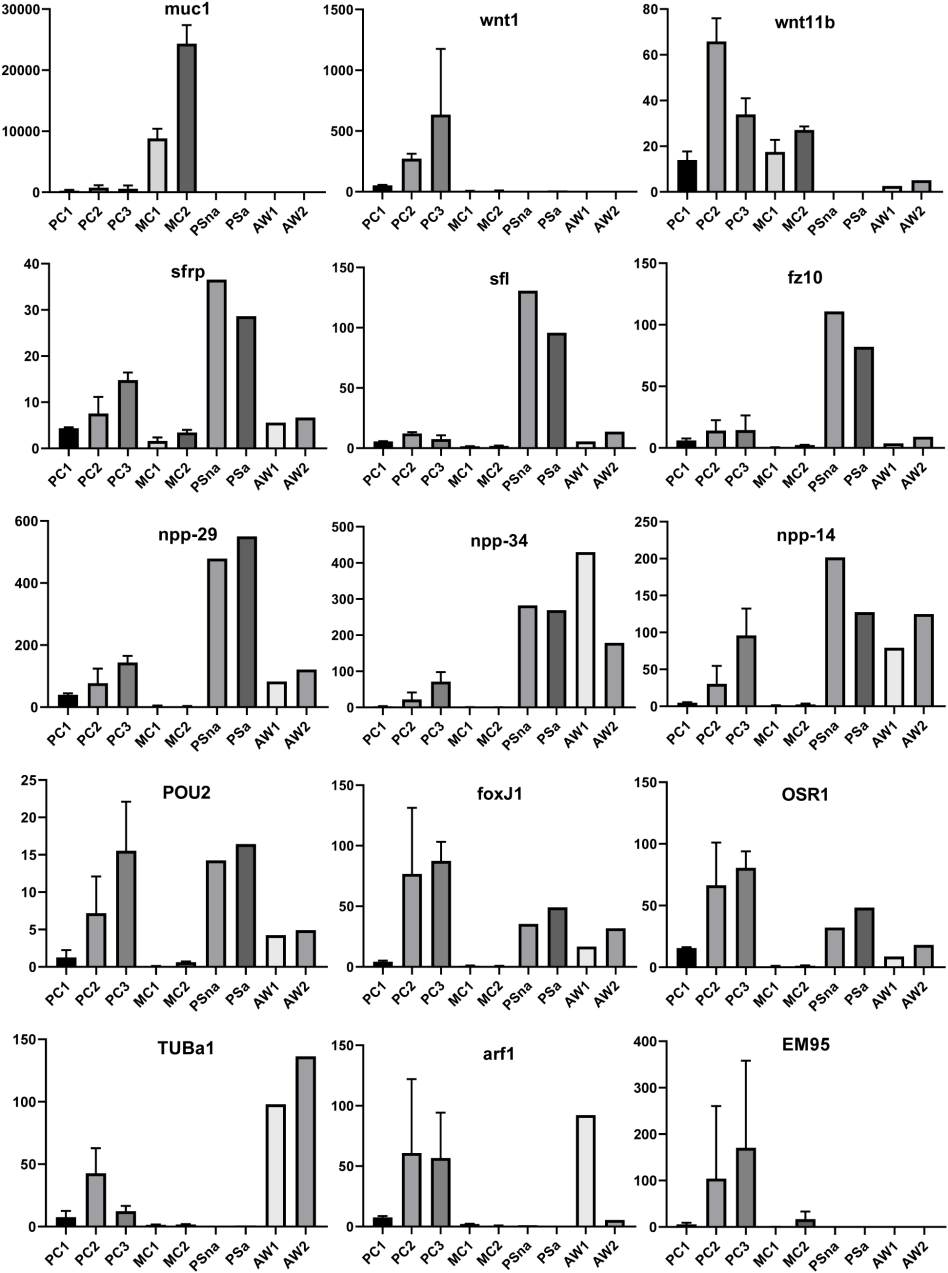
Expression of selected genes in *Echinococcus* cell culture, larvae, and adult worms. Displayed are expression values (TPM) in primary cell cultures after 2 d (PC1), 7 d (PC2), and 11 d (PC3) of *in vitro* cultivation as well as expression in cultivated metacestode vesicles, which served as controls for HU treatment (MC1) and Bi2536 treatment (MC2). Shown are mean values ± standard deviation of biological triplicates (n = 3). For comparison, TPM values (n = 1) of non-activated (PSna) and activated protoscoleces (PSa) as well as pre-gravid (AW1) and gravid (AW2) adult worms are shown. Individual genes are indicated above.

**Figure 6:**
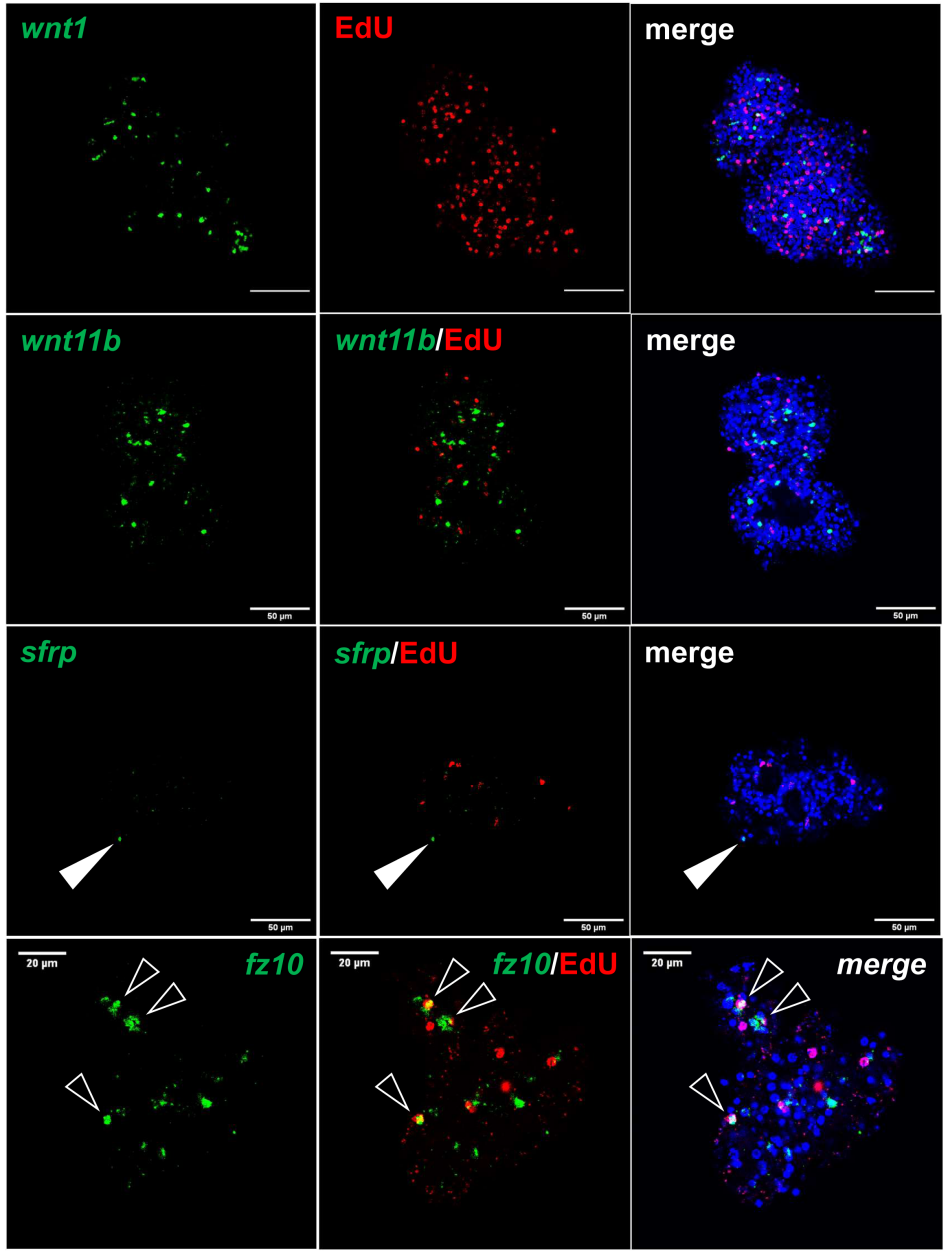
Expression of *Echinococcus* genes in primary cell cultures. WISH has been carried out on 7 d old primary cell cultures for selected genes (as indicated). Shown are in each panel from left to right of single confocal slices: WISH signal (green), WISH plus EdU (red, S-phase GC), and WISH plus EdU plus DAPI (nuclei, blue). Closed white triangle indicates *Emsrfp*+ cell, open white triangles indicate cell double positive for EdU and *Emfz10*. Size markers represent 50 μm for *wnt1*, *wnt11b*, *sfrp*, and 20 μm for *fz10*.

We previously showed that the *E. multilocularis* metacestode is posteriorized tissue and contains numerous cells expressing posterior morphogens encoded by genes such as *Emwnt1* or *Emwnt11b* (Koziol et al., 2016a) and, as expected, we also observed high levels of *Emwnt1* and *Emwnt11b* expression in primary cell aggregates, particularly for stages PC2 and PC3 (**Figure 5**; **Supplementary Table S2**). WISH analyses then confirmed that PC2 stage primary cell aggregates display numerous *wnt1*+ and *wnt11b*+ cells (**Figure 6**), probably leading to a general posteriorized development of the emerging structures. Similarly, we found high expression in primary cell cultures of *Em-alp2* (EmuJ_000393400), encoding a metacestode-specific alkaline phosphatase (Koziol et al., 2014), but not the related gene *Em-alp3* (EmuJ_000752700). The latter encodes an alkaline phosphatase isoform that is active in the excretory system of the protoscolex (Koziol et al., 2014), indicating that at least some protoscolex-specific cell types are not formed in primary cell preparations that derive from metacestode cells.

Upon closer inspection of primary cell gene expression profiles, however, we found numerous other genes that are typically expressed in protoscoleces in PC1 and, particularly, PC2 and PC3. To quantify this, we initially (and arbitrarily) defined protoscolex-specific genes as having an at least 10-fold higher expression level in protoscoleces when compared to metacestode vesicles. We then analysed previously generated datasets (n = 1) for non-activated and activated protoscoleces (Tsai et al., 2013), mapped them to the genome, and determined their average gene expression compared to the metacestode profiles characterized in the present study. Of all annotated *E. multilocularis* genes, we identified 872 as being at least 10-fold higher expressed in protoscoleces versus metacestode vesicles. Of these 872 genes, 304 (35%) were at least 3-fold higher expressed in PC1 versus metacestode vesicles. This number increased to 644 (74%) in PC2 and 697 (80%) in PC3 when compared to metacestode vesicles (**Supplementary Table S5**). As shown in **Figure 5**, the respective list of genes included *sfrp* and *sfl*, which are expressed at the anterior pole of protoscoleces upon brood capsule formation, but which are not expressed in metacestode vesicles that are free of brood capsules (Koziol et al., 2016a). In the case of *sfrp* we also carried out WISH on primary cell aggregates after 7 days of culture and found distinct *sfrp*+ cells. In the context of body axis determination, we also identified another gene, *Emfz10* (EmuJ_000085700), encoding a member of the frizzled family of GPCRs, which act as receptors for Wnt – ligands in determining body axis patterns, and which we previously identified as a potential target for the *Echinococcus* micro-RNA *mir-71* (Pérez et al., 2019). *Emfz10* was expressed to relatively high levels of 82 TPM in activated and 110 TPM in non-activated protoscoleces but had only low expression (below 1 TPM) in both metacestode preparations. In PC1, on the other hand, *Emfz10* expression was already increased to ~6 TPM and further increased to ~14 TPM in PC2 and PC3. As shown in **Figure 6**, we then also identified numerous *Emfz10*+ cells, which partly co-localized with EdU signals, in primary cell aggregates by WISH. Interestingly, among the list of genes typically expressed in protoscoleces, but also in primary cell aggregates, we also found several that we previously showed to encode neuropeptides (*npp-29*, *npp-34*, *npp-14*; Koziol et al., 2016b), as well as another POU-domain containing transcription factor (*EmPOU2*), a forkhead-box transcription factor (*Em-foxJ1*), and an ortholog of the odd-skipped-related family of transcription factors (*EmPSR1*), which participate in body axis formation (**Figure 5**).

Since primary cell aggregates contained numerous cells typically found in the protoscolex, we extended our analyses to additional developmental stages, such as adult worms and oncospheres. Again, we mined available datasets (n = 1) for pre-gravid and gravid adult worms (Tsai et al., 2013) and searched for genes with prominent expression in adult forms, but low expression in protoscolex and metacestode. Several of those, like a gene encoding an α-tubulin isoform (*EmTUBa1*; EmuJ_000042200) or an ADP ribosylation factor (*Emarf1*; EmuJ_000674900) again showed relatively high expression levels in primary cells after 7 or 11 days of incubation (**Figure 5**). Finally, in previous transcriptomic analyses, one gene encoding an EM95 antigen isoform, EmuJ_000368620, was shown to be highly upregulated in activated oncospheres but had low expression in metacestode tissue (Huang et al., 2016). As shown in **Figure 5**, this gene displayed little or no or expression in protoscoleces, adult worms, or metacestode vesicles, but its expression levels in primary cells strongly increased from PC1 (5 TPM) to PC2 (104 TPM), then PC3 (170 TPM).

Taken together, our analyses on *E. multilocularis* primary cell cultures revealed they are initially highly enriched with stem cells (Koziol et al., 2014) that proliferate and differentiate into several distinct cell types, such as muscle- and nerve cells, as well as *Em-muc-1* expressing tegumental cells that surround internal outside-in cavities. From these cavities, mature metacestode vesicles later emerge. As expected, primary cell development appears to be skewed towards posteriorized cell fates under the influence of *wnt1* and *wnt11b* expressing cells, which later leads to fully mature, posteriorized metacestode vesicles. However, *E. multilocularis* primary cell cultures also produce, at least to a certain degree, cell types that typically occur in other developmental stages such as oncospheres, protoscoleces, and adult worms. These data indicate that metacestode derived stem cells initially used to set up primary cell cultures are not pre-determined to exclusively form metacestode progeny but are also capable of developing into other cell types from different life cycle stages.

### *E. multilocularis* primary cell cultures form cells expressing a target for praziquantel

**Figure 7:**
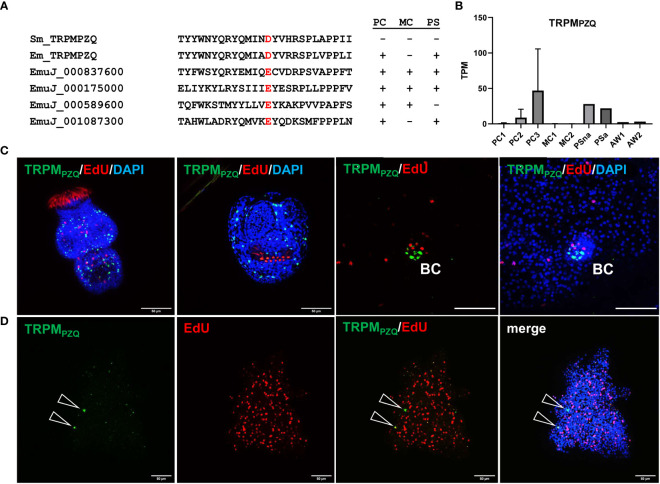
Sequence features and expression of EmTRPM_PZQ_. **(A)** Amino acid sequence comparison of different transient receptor potential calcium channels around the TRP domain amino acid residue responsible for PZQ sensitivity (highlighted in red). Displayed are sequences of TRPM_PZQ_ of *S. mansoni* (Sm_TRPMPZQ) and *E. multilocularis* (Em_TRPMPZQ; EmuJ_000986600) as well as different cation channels predicted in the *E. multilocularis* genome (indicated by gene ID). Expression levels of respective genes in primary cell cultures (PC), metacestode vesicles (MC), and protoscoleces (PS) are indicated to the right (+ = above 10 TPM, - = below 10 TPM). **(B)** Expression levels (in TPM) of *E. multilocularis* TRPM_PZQ_ in primary cell cultures, metacestode vesicles, protoscoleces, and adult worms according to RNA-Seq data (refer to **Figure 5** for abbreviations). **(C)** WISH for *E. multilocularis* TRPM_PZQ_ expression in protoscoleces and brood capsules. From left to right: activated protoscolex, invaginated protoscolex, metacestode tissue with brood capsule (BC; red, EdU, S-phase GC; gren, WISH, TRPM_PZQ_), metacestode vesicle with brood capsule (channels red, green, blue = DAPI, nuclei). Note the absence of signals in germinal layer. **(D)** Expression of TRPM_PZQ_ in *Echinococcus* primary cell aggregates. WISH of 7 d old aggregate showing from left to right: green channel (WISH, TRPM_PZQ_), red channel (EdU, S-phase GC), combined green and red channel, and merge of all channels (including blue, DAPI, nuclei). Open triangles indicate cells expressing TRPM_PZQ_. Size bar represents 50 μm for protoscoleces and primary cells, 20 μm for brood capsule. WISH images are from single confocal slices.

To further characterize the capability of *Echinococcus* primary cell cultures to generate cells that are typically not present in metacestode vesicles, we concentrated on a transient receptor potential (TRP) ion channel that has recently been identified as a target for praziquantel (PZQ) in the trematode *S. mansoni* (Park et al., 2019). Related ion channels are expressed by PZQ sensitive trematode and cestode species, and the presence of an Asp residue (instead of Glu) within the TRP domain is critical for PZQ binding (Rohr et al., 2023). The latter’s study authors suggested the ion channel encoded by EmuJ_000986600 as a likely target candidate for the activities of PZQ on adult *E. multilocularis* worms (Rohr et al., 2023). We therefore mined the available *E. multilocularis* genome information for orthologs to schistosome TRPM_PZQ_ and closely analysed those showing an expression level of 10 TPM or higher in protoscoleces or metacestode vesicles. As shown in **Figure 7**, the product of EmuJ_000986600 was the indeed the only TRP ion channel fulfilling these criteria and displaying an Asp residue within the TRP domain. We thus designated the respective protein EmTRPM_PZQ_.

Transcriptome analyses indicated that the gene encoding EmTRPM_PZQ_ is well expressed in protoscoleces but only lowly (<1 TPM) in metacestode vesicles (**Figure 7**; **Supplementary Table S3**), which agrees with the differential activities of PZQ against different *Echinococcus* larval stages (Taylor et al., 1989). We then performed WISH analyses for the EmTRPM_PZQ_ encoding gene (**Figure 7**), obtaining intense signals for activated and dormant protoscoleces. Interestingly, although we found the gene expressed in cells within developing brood capsules, no signal was obtained for the germinal layer of metacestode vesicles outside of developing protoscoleces (**Figure 7**), indicating that the EmTRPM_PZQ_ encoding gene is indeed not active in the metacestode. In primary cell culture aggregates, on the other hand, we identified few, but clearly detectable EmTRPM_PZQ_+ cells, again showing that this culture system forms cells which are typically found in larval stages other than metacestode vesicles.

### Host TNFα supports the development of metacestode vesicles from primary cells

**Figure 8:**
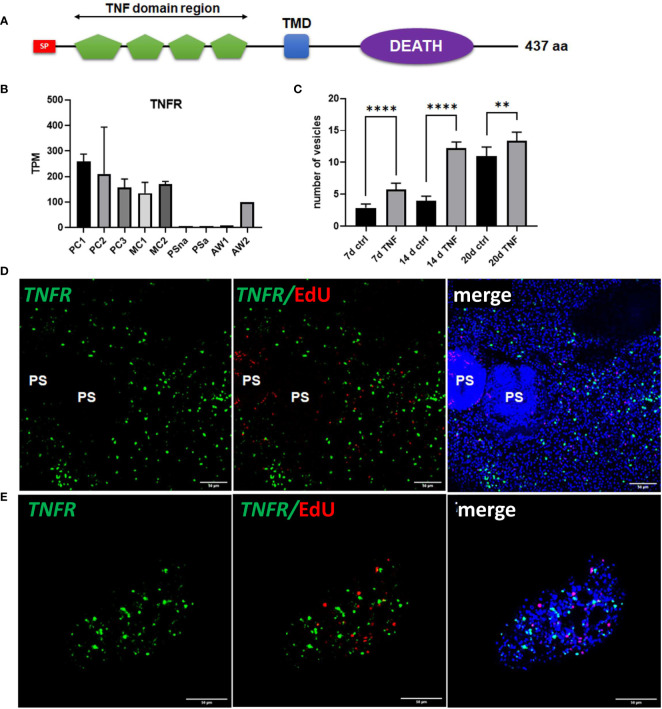
Sequence features and expression of *EmTNFR* as well as effects of TNFα on primary cell cultures. **(A)** Domain structure of EmTNFR. Displayed are the relative location of the following domains: signal peptide (SP, red), TNF-domains (green), transmembrane domain (TMD, blue), and DEATH domain (violet). **(B)** Expression (in TPM) of *EmTNFR* in primary cell cultures, larvae, and adult worms according to RNA-Seq data. Abbreviations as in **Figure 5**. **(C)** Effect of TNFα on the formation of metacestode vesicles from primary cell cultures. Primary cells have been incubated with (TNF) or without (ctrl) 10 ng/ml (43 nM) recombinant human TNFα for 7, 14, and 20 d (as indicated). The formation of mature vesicles has been counted. Displayed are median values ± standard deviation. Values after 7 d, 14 d, and 20 d have been compared using unpaired t-test. P-values are indicated by **** < 0,0001 and ** < 0,003. Experiments were performed in the biological triplicates with three technical triplicates. **(D)** WISH for *EmTNFR* on metacestode vesicles. Indicated are from left to right: green channel (WISH, *EmTNFR*), green and red channel (EdU, S-phase GC), merge of green and red channel with DAPI (blue, nuclei) of single confocal slices. Note the absence of signal in developed protoscoleces (PS). **(E)** Expression of *EmTNFR* in 7 d primary cell cultures. Channels are the same as in **(D)**. Size bar represents 50 μm in all images.

Since we already found high expression of posteriorizing factors, such as *wnt1* and *wnt11b*, within primary cell aggregates (**Figure 6**), which most probably direct the development of these cultures towards metacestode vesicles, we sought additional genes supporting these functions. By inspecting primary cell aggregate gene expression profiles we found one gene (EmuJ_000990500) with expression levels over 150 TPM in PC1, PC2, and PC3, which otherwise was highly expressed in metacestode vesicles but not in activated or dormant protoscoleces (**Figure 8**). According to the annotation on WormBase Parasite, EmuJ_000990500 encoded a member of the tumour necrosis factor (TNF) receptor superfamily, but by inspecting mapping reads around the gene locus we found the gene wrongly predicted, missing 3’ coding information. Based on the available genome information, we then fully cloned the respective cDNA and found that it encoded a protein of 437 amino acids with a predicted signal peptide, four TNF domains, a transmembrane domain, and an intracellular DEATH domain (**Figure 8**), which are all hallmarks of the TNF receptor family (Wallach, 2018). In BLASTP analyses against the SWISSPROT database, the encoded protein displayed highest homologies to different mammalian members of the TNF receptor family and to a previously characterized TNF receptor of the related trematode *S. mansoni* (Oliveira et al., 2009). We thus named the gene *Em-tnfr*, encoding the protein Em-TNFR.

We then carried out *Em-tnfr* specific WISH on metacestode vesicles and found *Em-tnfr*+ signals distributed over the entire germinal layer with no co-localization with EdU, indicating that the gene is exclusively expressed in post-mitotic cells. Interestingly, no signals were detected in brood capsules or developed protoscoleces (**Figure 8**), which is in line with the transcriptome data (**Supplementary Table S2**) and indicates that *Em-tnfr* is a metacestode specific gene (**Figure 8**). We then also carried out combined EdU/WISH analyses on primary cell preparations and found numerous *Em-tnfr*+ (but EdU-) cells dispersed throughout all aggregates, indicating that the gene is expressed by a dominant fraction of differentiated cells within the developing cultures (**Figure 8**). Since the orthologous TNFR of *S. mansoni* is considered to serve as a receptor for host TNFα (Oliveira et al., 2009; Lopes-Junior et al., 2022) and since TNFα is one of the dominant cytokines regulating early immune responses during AE (Gottstein et al., 2015), we then investigated whether mammalian TNFα could stimulate metacestode development by primary cells. To this end, primary cell cultures were incubated with physiological concentrations (10 ng/ml; 43 nM) of recombinant human TNFα. As depicted in **Figure 8**, after 7 and 14 days, human TNFα highly significantly accelerated the formation of mature metacestode vesicles from primary cells, indicating that the host immune response during early AE may have a beneficial effect on the formation of the metacestode.

Taken together, these analyses demonstrated that the gene expression profile of primary cell cultures is dominated by factors supporting the development of stem cell progeny towards posterior fates and metacestode vesicles, although to a certain degree also cells are produced which are typical for protoscoleces, adult worms, and oncospheres. In addition to host insulin (Hemer et al., 2014) and fibroblast growth factor (Förster et al., 2019), we herein identified a third host cytokine, TNFα, which accelerates the formation of metacestode vesicles by primary cells in culture. Whether these effects are mediated by direct stimulation of EmTNFR through TNFα remains to be established by detailed biochemical and cell biological studies.

## Discussion

AE is a stem cell driven disease because proliferation and development of the infiltratively growing metacestode stage is exclusively mediated by neoblast-like GC, which are the only mitotically active cells within metacestode tissue and give rise to all differentiated cells (Koziol et al., 2014). It is thus obvious that efforts towards the development of novel anti-infectives against AE must target the GC population and it has already been suggested that the limited efficacy of current chemotherapeutic treatment against AE is due to reduced activity of albendazole or mebendazole against the parasite’s GC (Brehm and Koziol, 2014; Koziol and Brehm, 2015). We herein describe the first characterization of GC associated genes using targeted transcriptomic techniques, validated with several complimentary approaches. We used *in vitro* cultivated metacestode vesicles that were specifically deprived of GC by two different methods previously shown to have minimal unspecific effects, at least on the overall morphology of parasite vesicles as well as on muscle cells, nerve cells, and the tegument (Koziol et al., 2014; Schubert et al., 2014). The set of ~1,180 genes with a significant reduction of transcripts in both settings, was then tested for enrichment in parasite primary cell cultures, which are strongly enriched in GC (Koziol et al., 2014), and indeed 90% of identified genes also hadhigher expression under these conditions. Further, we used RT-qPCR and WISH in combination with EdU incorporation to verify our results for a selection of genes. We inspected the list of GC associated genes for plausibility, and found that all genes previously shown to be expressed in significant subsets of GC were present in the list, whereas genes known to be mainly expressed in differentiated cells were absent. We are thus confident that the list of GC associated genes presented in our study provides a robust overview of *E. multilocularis* factors associated with stem cells in one of the following ways: exclusively expressed in GC; expressed in GC and differentiating progeny; or exclusively expressed in differentiated cells, but requiring the presence of GC for transcription. It should also be noted that for several of the factors in our list of GC associated genes, functional analyses already showed an involvement in stem cell driven *Echinococcus* developmental processes. Pharmacological inhibition of EmPlk1 (EmuJ_000471700), EmMPK3 (EmuJ_000174000), PIM kinase (EmuJ_000197100), or Aurora kinases (EmuJ_000891900; EmuJ_001059700), for example, has led to clear reductions of GC in metacestode vesicles (Schubert et al., 2014; Cheng et al., 2019; Stoll et al., 2021; Koike et al., 2022), implying a role of the respective genes in stem cell maintenance.

To achieve maximum depletion of stem cells prior to transcriptome analyses we performed metacestode vesicle treatment for 7 days with HU and for 21 days with Bi-2536, which most likely exceeds the average cell cycle duration of GC in metacestode vesicles (Koziol et al., 2014; Cheng et al., 2017a). It is thus possible that our list of GC associated factors also contains genes that are not expressed in GC, but in GC progeny already committed to a specific cell fate. The presence of committed cells could explain the differences in gene numbers with reduced read counts in Bi-2536 treated vesicles (2,592 genes; 21 days treatment) versus HU treated vesicles (1,788 genes; 7 days); indeed, the restriction of factors like EmuJ_000495700 to the list of genes affected by Bi-2536 treatment, but not by HU treatment, indicates that this could be the case. EmuJ_000495700 is predicted to encode a MEX3B-like RNA binding protein, homologous to planarian MEX3-1 that is required for differentiation during stem cell lineage development and is exclusively expressed in stem cell progeny (Zhu et al., 2015). Furthermore, after Bi-2536 treatment, but not after HU treatment, we observed significant read reductions for genes like *Em-sert* (serotonin transporter; EmuJ_000391300) or *Em-wnt1* (EmuJ_000349900), for which we previously reported predominant expression in nerve or muscle cells, respectively (Koziol et al., 2016a; Herz and Brehm, 2021). In both cases it is conceivable that these genes are not exclusively expressed in terminally differentiated cell types, but also in GC progeny committed to neuronal or muscular development. For our definition of GC associated genes, we thus consider it justified to concentrate on gene expression profiles of HU treated vesicles, thus minimizing the possibility of false positives that are mainly expressed in GC progeny.

Of course, by restricting the list of genes to those that are significantly reduced under both GC eliminating strategies, we will miss some factors that are expressed in a GC associated manner but are affected either only by HU- or by Bi-2536 vesicle treatment. For example, several genes with significantly reduced read counts in Bi-2536 treated vesicles, but not in HU-treated vesicles, encode orthologs to haspin kinase (EmuJ_000667600), the cell cycle checkpoint protein RAD17 (EmuJ_000702400), or the structural maintenance of chromosomes protein 4 (EmuJ_000517500) with presumed functions in cell cycle control, mitosis and/or replication. Overall, we thus consider the list of 1,180 GC associated genes presented herein a very conservative estimation of the *Echinococcus* stem cell gene expression profile and propose that additional GC associated genes might be found among the factors that show reduced read counts upon either HU- or Bi-2536 treatment. Furthermore, we suggest that our list of genes with significantly diminished read counts after Bi-2536 treatment is enriched with factors that play important roles in the differentiation of GC progeny.

It should be emphasized that we concentrated on the gene expression profile of metacestode stem cells. Given that the metacestode represents posteriorized tissue (Koziol et al., 2016a) it is conceivable that GC that are localized at anterior regions within the protoscolex, express additional genes. Alternatively, adult worm GC may express additional factors that are not included in our list. One possible example is the protoscolex specific gene *Emfz10* that is expressed in primary cell cultures. *In situ* hybridization showed that *Emfz10* is expressed in many EdU+ cells during primary cell development (**Figure 6**), nevertheless this gene is not contained in our list of GC associated genes, most probably because its expression level within metacestode tissue is too low to yield statistically significant reduction upon HU- and Bi-2536 treatment. Although we are confident that by incorporating metacestode vesicles and primary cells into our analyses, we cover the vast majority of stem cell associated *Echinococcus* factors in our study, an even more comprehensive dataset would be obtained by carrying out complementary studies on protoscoleces. Following the strategy pursued herein, transcriptome comparisons between activated protoscoleces after HU- and Bi-2536 treatment would be one way to characterize these additional factors.

Our previous analyses revealed that *Echinococcus* GC significantly differ from other stem cell systems, including planarian neoblasts and schistosome stem cells, in gene expression profiles (Koziol et al., 2014; Förster et al., 2019). First, although the *Echinococcus* genome contains *nanos* orthologs, which are important stem cell markers in schistosomes (Wang et al., 2018) and necessary for regeneration in planarian germ cells (Wang et al., 2007), only very small subsets of GC express *Em-nos-1* and *Em-nos-2* (Koziol et al., 2014). In both planarians and schistosomes, fibroblast growth factor (FGF) receptor genes have already been identified as important stem cell markers (Ogawa et al., 2002; Wang et al., 2018). Related receptors have been identified in *Echinococcus* but only one of these, *emfr3*, is expressed in stem cells (and part of our list of GC associated genes; EmuJ_000893600) and, even there, only in a very small sub-population (Förster et al., 2019). Finally, cestodes, like the related schistosomes, have lost classical stem cell markers such as *piwi* and *vasa* but instead evolved different clades of *piwi*-like Argonaute and *vasa*-like DEAD-box helicase genes (PL10), which might fulfil related tasks in stem cells (Tsai et al., 2013; Skinner et al., 2014). In schistosome sporocysts, the *ago2-1* gene is expressed in all neoblast-like stem cells (Wang et al., 2018), whereas in *Echinococcus* (as in planarians) both *ago* genes appear to be ubiquitously expressed, as previously demonstrated by us (Koziol et al., 2014) and confirmed in the present study. At least in planarians, a *piwi* ortholog (*smedwi-1*) serves as a general marker of neoblasts (Molina and Cebrià, 2021) and in schistosomes DEAD-box helicases of the PL10 family have important germline functions (Skinner et al., 2020). Most notably, however, neither of the two *PL10* genes encoded by the *Echinococcus* genome (EmuJ_000098400; EmuJ_001183300) is present in our list of GC associated genes. Although both factors are expressed highly in primary cell cultures (**Supplementary Table S2**), they do not show reduced read numbers after either HU or Bi-2536 treatment, indicating that they are ubiquitously expressed. All these differences support the uniqueness of the *Echinococcus* stem cell system and indicate that it might have arisen as an adaptation to the asexual amplification mode within the intermediate host. For a closer characterization of GC sub-populations with differing proliferative potential and fate, it will be important for future investigations to carry out single cell sequencing of isolated metacestode cells, combined with WISH and pulse-chase experiments on cultivated metacestode vesicles. Several of the genes that we characterized in the present study will be highly useful in these efforts, particularly *EmCIP2Ah* as a general marker for GC and *Emkal1* as a possible marker for a GC subset with prolonged cell cycle.

The analyses we carried out concerning the *E. multilocularis* primary cell cultivation system clearly indicated that GC, after isolation from metacestode vesicles, have the potential to develop into various directions and are not confined to the production of metacestode specific cells. Based on previous analyses showing that 2 day old primary cell cultures are enriched in GC (80%) but also contain muscle and nerve cells (Koziol et al., 2014), that muscle cells of the GL express posteriorizing position control genes such as *wnt1* and *wnt11b* (Koziol et al., 2016a), and that position control information is also released by germinal layer nerve cells (Kaethner et al., 2023), we propose that differentiation processes in GC of primary cell aggregates are strongly influenced by posteriorizing morphogens that are released by the co-cultured muscle and nerve cells (which also derive from metacestode vesicles). We suggest that, in this environment of high Wnt1/Wnt11b the majority of GC are directed towards posterior fates and, upon proliferation and differentiation, produce additional Wnt1 and Wnt11b releasing muscle cells alongside nerve cells that are typically encountered in metacestode vesicles. This would explain why in our transcriptional analyses we observed a continuous decrease of stem cell specific gene expression from PC1 to PC2 and PC3 (through a relative decrease of the proportion of undifferentiated GC), and a continuous increase of posteriorizing positional control gene expression (simply by increasing numbers of differentiated cells). In this model, it would therefore be the influence of posteriorizing morphogens released by metacestode-derived muscle and nerve cells that drives most stem cells towards metacestode vesicle production, and not an intrinsic predisposition of metacestode-derived stem cells to themselves develop towards metacestode tissue.

This model would also explain why some GC in primary cell cultures form cells that are not metacestode typical. Within regular metacestode vesicles the widespread distribution of Wnt1 and Wnt11b producing cells most likely ensures that all GC are subject to a posteriorizing environment until locally low Wnt1/Wnt11b conditions are induced, resulting in the formation of brood capsules (Kaethner et al., 2023). Since the architecture of primary cell aggregates differs significantly from metacestode vesicles, the posteriorizing effect of Wnt1 and Wnt11b most probably does not reach all stem cells, which then results in differentiation processes that are more typical for cells within brood capsules, protoscoleces, or even adult worms and oncospheres. This would explain why cells that express atypical metacestode genes are mostly located in exterior regions of primary cell aggregates, whereas *wnt1* and *wnt11b* expressing cells are more centrally located (**Figure 6**, **Figure 7**). Again, this model implies that *Echinococcus* GC which derive from metacestode vesicles are not pre-determined to a specific fate, but dynamically respond to the environment of positional information to which they are exposed. It remains an open question whether in the absence of a high Wnt1/Wnt11b environment *Echinococcus* GC randomly produce differentiated cells as some kind of default mechanism, or whether they locally follow directed development towards anterior fates within primary cell aggregates. Our transcriptional analyses at least indicate that anteriorizing morphogens such as *sfrp* and *sfl* are expressed within primary cell aggregates, indicating that ‘head organizing’ structures are present. Considering this model, it would also be worthwhile to re-visit previous studies concerning the influence of host hormones and cytokines on primary cell development. We had, for example, previously observed that *Echinococcus* primary cells produced significantly more mature metacestode vesicles when incubated with host derived FGF (Förster et al., 2019). This could be due to a general stimulation of stem cell proliferation, which was indeed observed in intact metacestode vesicles in response to FGF (Förster et al., 2019). However, at least in planarians FGF appears to antagonize the anteriorizing effects of *nou darake*, which encodes a soluble form of FGF receptors (Cebrià et al., 2002). An ortholog to *nou darake* is also encoded by the *Echinococcus* genome (EmuJ_000770900) and is well expressed in primary cell cultures (**Supplementary Table S2**). Hence, provided that the mechanisms of head formation are comparable between *Echinococcus* and planarians, which is highly likely (Koziol et al., 2016a), the stimulating effects of host FGF on metacestode vesicle production could also be due to an inhibition of anteriorizing activities within primary cell aggregates, thus leaving more stem cells for posteriorized development. Such a mechanism could also explain the effect of host TNFα on vesicle production by primary cells. At least in some experimental settings concerning mammalian cells, TNFα clearly stimulates malignant transformation or osteogenic differentiation by inducing the Wnt signaling pathway (Li et al., 2020; Zhao et al., 2020). Further experiments are necessary to unravel the precise biochemical mechanisms by which TNFα leads to enhanced vesicle formation. Our data at least indicate that the early immune response during AE, which is characterized by a high TNFα environment (Gottstein et al., 2015), could stimulate oncosphere derived GC to produce mature metacestode vesicles.

The complex genetic network that regulates Echinococcus GC self-renewal and differentiation will most likely involve many of the 44 transcription factor encoding genes identified herein as being expressed in a GC associated manner. For at least one of these genes, *EmSox2*, an important role in stem cell function has already been established (Cheng et al., 2017a) and for several of those factors, such as the orthologs to *foxD* and *tsh*, it is likely that they not only contribute to general stem cell differentiation processes, but also to mechanisms associated with the peculiar mode of *E. multilocularis* to suppress anterior development within the asexually growing metacestode. Deciphering the precise function of these genes in GC biology requires functional genomic methodology that, unfortunately, is still limited in the case of *Echinococcus*. One of the advantages of the primary cell culture system is that it is amenable to gene manipulation via RNA interference (Spiliotis et al., 2010; Pérez et al., 2019). As revealed by our transcriptomic analyses, the vast majority of GC associated genes, including all 44 transcription factors, are well expressed in primary cells. By a combination of RNAi methodology with *in situ* hybridization on GC marker genes and pulse-chase experiments concerning GC progeny it should thus be technically possible to approach functional analyses of GC associated genes. Respective experiments are currently underway in our laboratory as are approaches towards single cell transcriptomic analyses of metacestode cells, for which the GC markers identified in this study will be highly valuable.

## Data availability statement

The datasets presented in this study can be found in online repositories. The names of the repository/repositories and accession number(s) can be found in the article/**Supplementary Material**.

## Ethics statement

The animal study was approved by the Ethics Committee of the Government of Lower Franconia, Würzburg, Germany, under permit numbers 55.2–2531.01-61/13 and 55.2.2-2532-2-1479-8. The study was conducted in accordance with the local legislation and institutional requirements.

## Author contributions

MH: Data curation, Formal analysis, Investigation, Methodology, Visualization, Writing – original draft. MZ: Investigation, Validation, Writing – original draft. LW: Data curation, Investigation, Validation, Formal analysis, Visualization, Writing – original draft. KP: Data curation, Formal analysis, Investigation, Visualization, Writing – review & editing. RH: Data curation, Formal analysis, Investigation, Visualization, Writing – review & editing. CB: Formal analysis, Investigation, Visualization, Writing – review & editing. NH: Data curation, Formal analysis, Investigation, Writing – review & editing. TH: Data curation, Investigation, Validation, Writing – review & editing. MoB: Data curation, Investigation, Validation, Visualization, Writing – review & editing. MS: Formal analysis, Investigation, Writing – review & editing. UK: Conceptualization, Data curation, Formal analysis, Investigation, Methodology, Validation, Visualization, Writing – original draft, Writing – review & editing. MaB: Conceptualization, Data curation, Formal analysis, Funding acquisition, Supervision, Validation, Visualization, Writing – original draft, Writing – review & editing. KB: Conceptualization, Data curation, Formal analysis, Funding acquisition, Investigation, Methodology, Project administration, Supervision, Validation, Visualization, Writing – original draft, Writing – review & editing.
