# Supplementary material for: Genome-wide transcriptome analysis of Echinococcus multilocularis larvae and germinative cell cultures reveals genes involved in parasite stem cell function
Source: Front Cell Infect Microbiol. 2024 Jan 25;14:1335946. doi: 10.3389/fcimb.2024.1335946 (PMC10850878; doi:10.3389/fcimb.2024.1335946)
Supplement: Supplementary file 6 [file DataSheet_1.pdf]

Figure S1

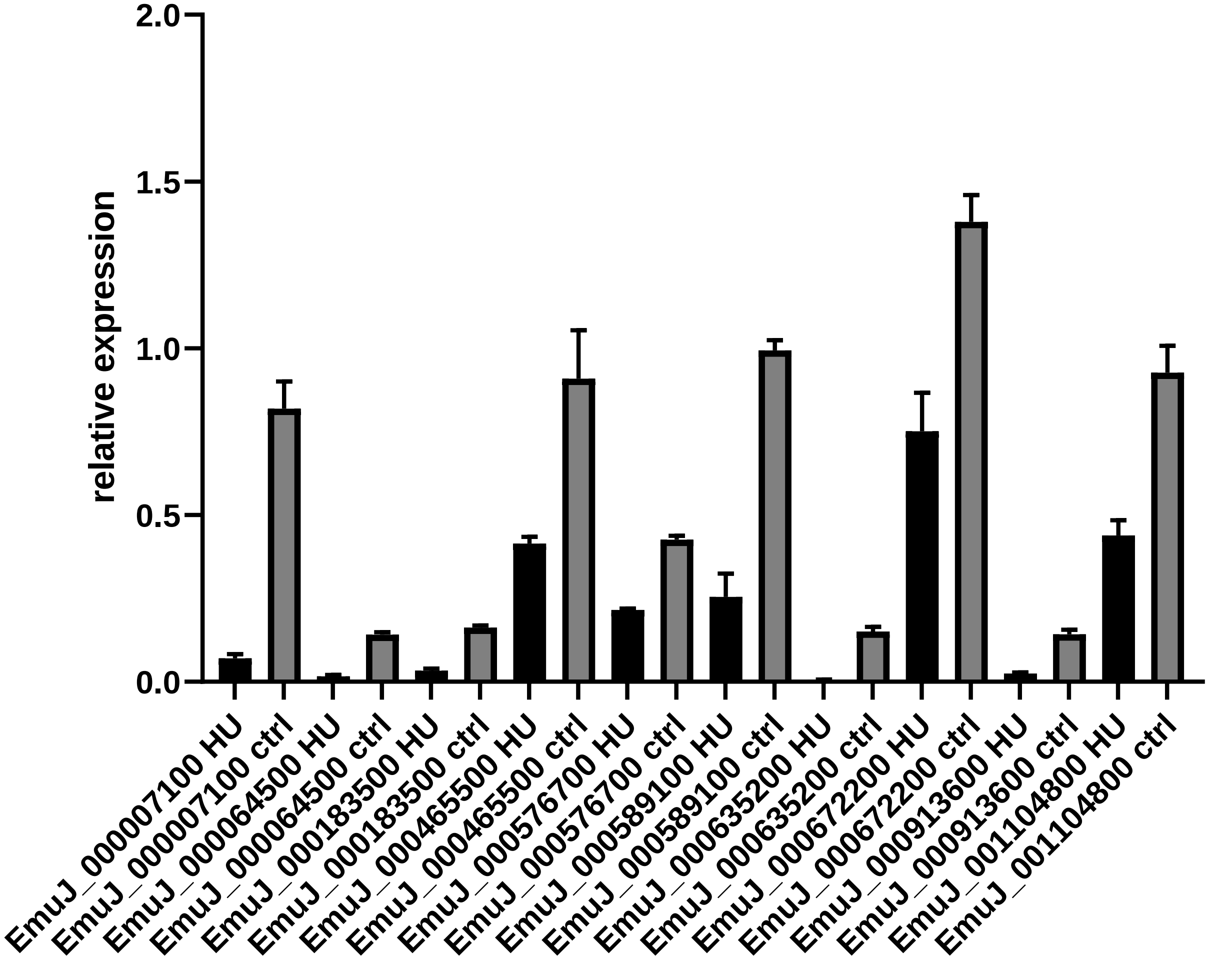

Figure S1. RT-qPCR analysis of selected GC associated genes. Depicted are expression values relative to the housekeeping gene *elp* (EmuJ_000485800). Indicated below the graph are Gene IDs and samples (HU = HU depleted vesicles; ctrl = control vesicles without HU treatment). All experiments were performed in biological triplicates. Error bars show standard deviation.
