# Supplementary material for: Genome-wide transcriptome analysis of Echinococcus multilocularis larvae and germinative cell cultures reveals genes involved in parasite stem cell function
Source: Front Cell Infect Microbiol. 2024 Jan 25;14:1335946. doi: 10.3389/fcimb.2024.1335946 (PMC10850878; doi:10.3389/fcimb.2024.1335946)
Supplement: Supplementary file 7 [file DataSheet_2.pdf]

Figure S2

Emhlh1 (EmuJ_000098000; OR233046)

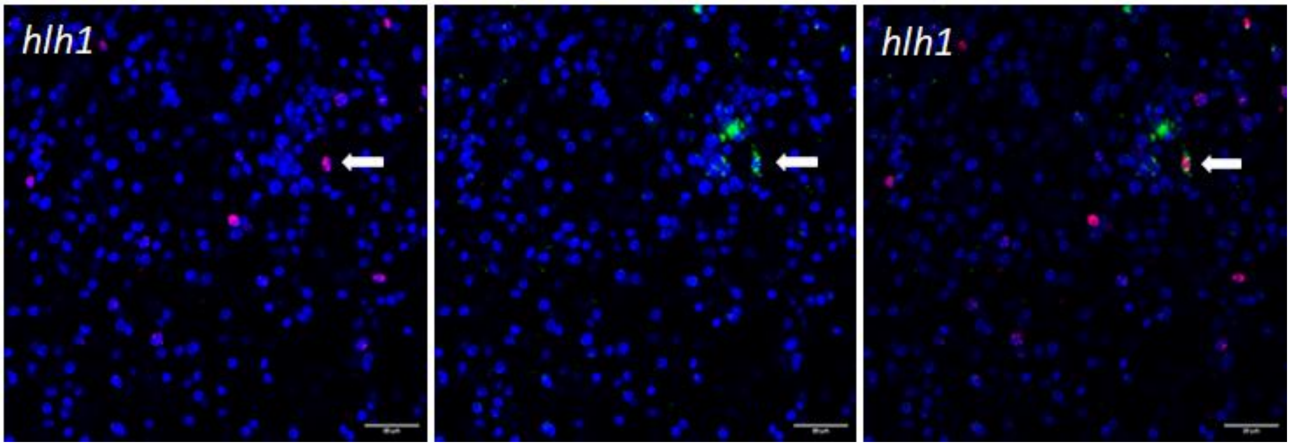

MVVLSSAYTAKSNSFSITNLLGDVNGKAATYSANLPRENR 40
ARVAQQRIKANARERGRVHTIGAAFEALRKSVPVSGECKL 80
TKLSVLRIAAAYIETLVACLEASRIEEQEEEAEAKEMEKP 120
EDLSRSARVSPNHLPSGATAVIRFNHCSQKLMKRIQRECR 160
GSVFHLS 167

```
ATOH8_Hs    NARERTRVHTISAAFEALRKQVPCYSYGQKLSKLAILRIACNYILSLARLADL 53
Emhlh1      NARERGRVHTIGAAFEALRKSVPVSG-ECKLTKLSVLRIAAAYIETLVACLE  51
            ***** *****.********.**  .   **:**::****. ** :*.   :
```

Figure S2A: Sequence features and expression of *Emhlh1*. Depicted are the *E. multilocularis* transcript ID numbers and GenBank accession numbers for *Emhlh1* (above). Upper panel: WISH analysis for *Emhlh1* on *in vitro* cultivated metacestode vesicles. Channel settings were blue (DAPI, nuclei), red (EdU, S-phase GC), and green (WISH+). Shown are from left to right: DAPI/EdU, DAPI/WISH+, and merge of DAPI/EdU/WISH+. White arrows mark cells double positive for EdU and WISH. Size bar = 20 µm. Middle panel: Deduced amino acid sequence of Emhlh1. The predicted helix-loop-helix domain is marked in blue. Amino acid sequence numbering indicated to the right. Lower panel: Amino acid sequence comparison between the helix-loop-helix domains of Emhlh1 and human atonal homolog 8 (ATOH8; GenBank accession no. Q96SQ7). Sites of perfect alignment (*) as well as groups of strong (:) or weak (.) similarity are marked below the alignment.

EmPOU1 (EmuJ_000449700; OR233047)

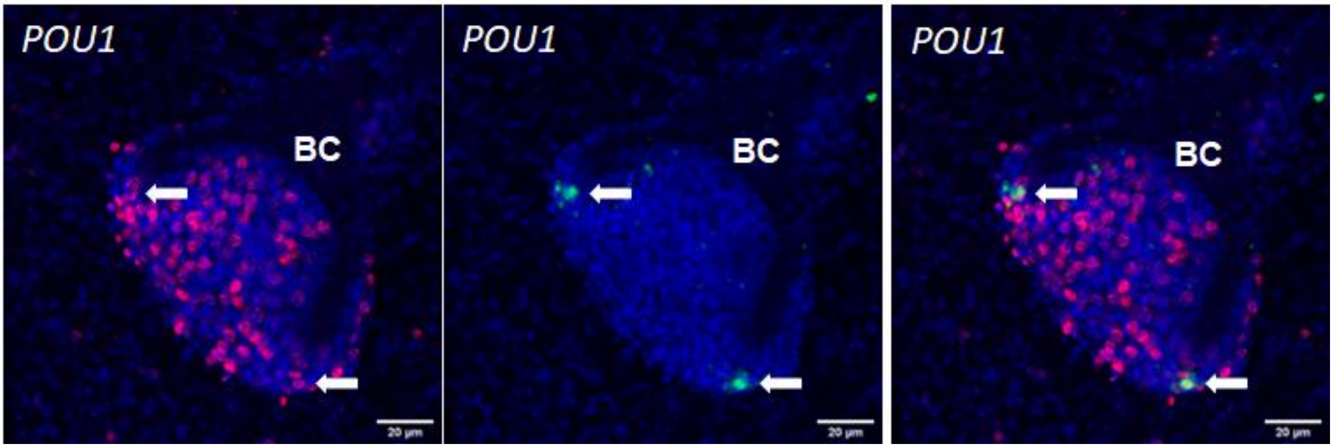


```
MICSDDPNQHPRHQSTIDFPSNQSPDSPKPAPPIPDGSNSSHGGRKRGHKKSNAFCHLEA 060
GAEEAEEVEIAYPVNSIAPEPAQQFVPSWQFEQPGFPVYATGHEAQYPGLFPPHQNFLAV 120
NHVLPWNPESDETWKSTSPFPPEAYTQFPTTEYNPCWSRSSQGTQSLIIEETKPDLLLYS 180
IPRSENVYETAFRGQTGESNQHTFQAPEVDEYIVQGDEMKPPYPFPLNGHLPLVHFQPED 240
HSTATISTSSLESDLNATNNSGSEPETKLNHLLRTAGIESLPLCFKDGEGWNTSPYIAPP 300
QVQHQPPPPPPPTNVANPLLSEQLSSVLPNTEVGNMKSPSEDALSTGFPLLPSFSLPAAY 360
RLIPPTETADFDLSQVGTASSPNSGGENLSQPQEEFPSSDDLEHFAKLFKQRRIKMGYTQ 420
ADVGLALGTLYGNVFSQTTICRFEALQLSFKNMCKLKPLLQKWLHEADCCTSTTSNFDKV 480
TTQGRKRKKRTSIEVGVKGVLEGHFIRQPKPAAQDITNLADTLGLEKEVVRVWFCNRRQK 540
QKRLNPAAFENGGDLSENSLRGGEGGSGYSTPPFCGGGADGTHQMVNSPFPNGGGFFHEN 600
PSALYFEGGFPQQSAVEACEYPSQAPPSQPPSHSSCLVLTTPPDYLLPHLQSVDKPPEVV 660
TPYPTATLPETAIYSV                                             676
```

```
CF1A_Dm      EDTPTSDDLEAFAKQFKQRRIKLGFTQADVGLALGTLYGNVFSQTTICRFEALQLSFKNM     60
EmPOU1       EEFPSSDDLEHFAKLFKQRRIKMGYTQADVGLALGTLYGNVFSQTTICRFEALQLSFKNM     60
             *: *:***** *** *******:*:***********************************

CF1A_Dm      CKLKPLLQKWLEEADSTTGSPTSIDKIAAQGRKRKKRTSIEVSVKGALEQHFHKQPKPSA     120
EmPOU1       CKLKPLLQKWLHEADCCTSTTSNFDKVTTQGRKRKKRTSIEVGVKGVLEGHFIRQPKPAA     120
             ***********.***. *.: :.:**:::*************.***.** ** :****:*

CF1A_Dm      QEITSLADSLQLEKEVVRVWFCNRRQKEKRMTPPN      155
EmPOU1       QDITNLADTLGLEKEVVRVWFCNRRQKQKRLNPAA      155
             *:**.***:* ****************:**:.*
```

Figure S2B: Sequence features and expression of *EmPOU1*. Depicted are the *E. multilocularis* transcript ID numbers and GenBank accession numbers for *EmPOU1* (above). Upper panel: WISH analysis for *EmPOU1* on *in vitro* cultivated metacestode vesicles. Channel settings were blue (DAPI, nuclei), red (EdU, S-phase GC), and green (WISH+). Shown are from left to right: DAPI/EdU, DAPI/WISH+, and merge of DAPI/EdU/WISH+. White arrows mark cells double positive for EdU and WISH. Size bar = 20 µm. BC = brood capsule. Middle panel: Deduced amino acid sequence of EmPOU1. Predicted POU (blue) and Hox (green) domains are marked. Lower panel: Amino acid sequence comparison between the POU-Hox domain regions of EmPOU1 and Drosophila chorion factor 1A (CF1A_Dm; GenBank accession no. P16241). Sites of perfect alignment (*) as well as groups of strong (:) or weak (.) similarity are marked below the alignment.

EmPSA1 (EmuJ_000356700; OR233048)

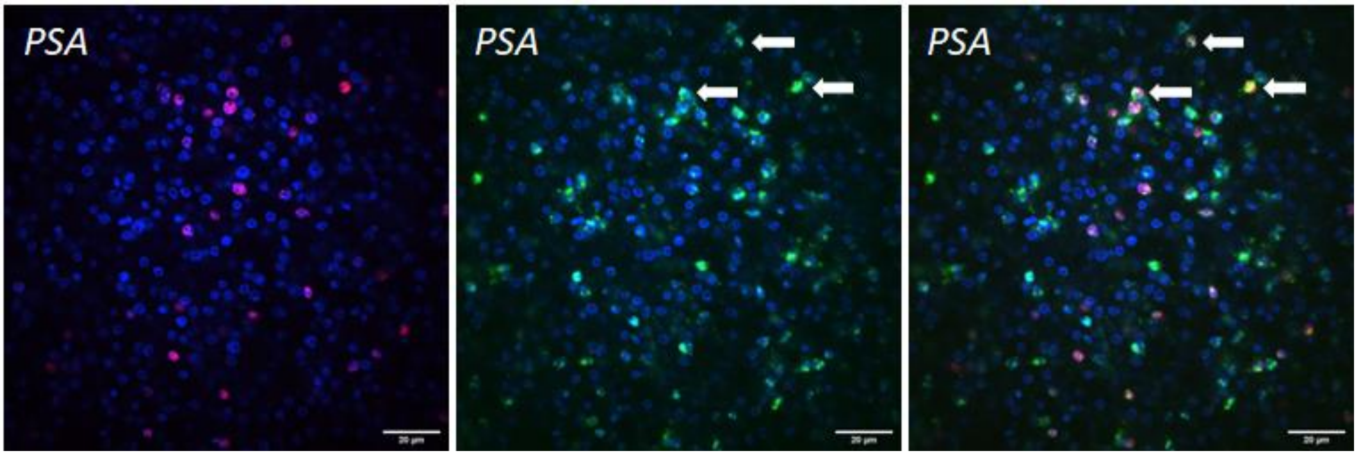


```
MAEDIARHYRLPRVVKPVNYRLDFVPNFSSLTFTARASVELQVIEPTNRIIFNSRGLEILSATYHDTRATITYDEEQEIV 080
VFGFPTELQSGKGYLNLDFHGEFANDMLGLYHSTYTDVMGKKYNIIATQFESVFARRAFPCMDEPDRKATFEISIVALDD 160
QVALSNMPEISRDVVPTPEGCSEPLDGHSYVKITFDRTPIMSTYIVAMVLGYFEYISATAPGNHISNVSSSSASETEEST 240
EALPPEVEIRVYTPLGKRDFGQHALTVVKKSLPFYAKLFGYPYPLPKLDLVAIPDFACNAMENWGLVTYRETALLIDPEN 320
SSLASKQNVALTVAHEVSHMWFGNLVTMSWWTDLWLNEGFATWAEYLAVDHCFPDYDIWTLFVSREYIRALQLDELKSSH 400
PIQVEVNSAREVEEIFDAVSYQKGSCVIRMLYNYVGASSFEAGLKSYFKRFKYSNAETQDLWTALEATGVDNLTELMSPW 480
TKQTGYPVLSVRRICAPDGTYSIGLKQQRFLADSSSTEEESLVCWRIPIDVCAVDDSKSILFRLVTDIPSPSPYSTKTPE 560
HGDKEQFTPETGVKSQEIIHFLPDSDLSPRVRLNPNAIGFYRVHYDSAMMDTILEAISRGTVPERDRVSLLDDQFALARA 640
GFQGLDKVLQFCRAFVGETRYSVWSVLSERLAQVQTLLEEASYPAEDEVVFPEASREICGLNNLYMELALPVYEKIGFKP 720
IPYESNNDRLLRSIIISILGRIGHSDVITNARTAFTRHHAAVISASVGEAAVDQSNLISPDLRTAVYSICMRSGGDKEFW 800
KLFELYNQATLNDERVRILSSLGATTNADIIQRVFKLTFTEDVRKQDRFHVLLSVTGSAGGRRALWNLVRARIATLSEDL 880
GTSHLLARVLVGSASSFALQERYDEIKAFYEEHDVPCPRVIQQTLEAVKINVAQWKRDEKAVSTFLNNLADKDKPISPSH 960
HVSPKRRRLAPSNALKEKNLP                                                            981
```

```
Hs_PSA      DGVCVRVYTPVGKAEQGKFALEVAAKTLPFYKDYFNVPYPLPKIDLIAIADFAAGAMENW      60
EmPSA1      PEVEIRVYTPLGKRDFGQHALTVVKKSLPFYAKLFGYPYPLPKLDLVAIPDFACNAMENW      60
              * :*****:** : *:.** *. *:**** . *. ******:**:** ***..*****

Hs_PSA      GLVTYRETALLIDPKNSCSSSRQWVALVVGHELAHQWFGNLVTMEWWTHLWLNEGFASWI      120
EmPSA1      GLVTYRETALLIDPENSSLASKQNVALTVAHEVSHMWFGNLVTMSWWTDLWLNEGFATWA      120
            **************:**. :*:* ***.*.**::* ********.***.********:*

Hs_PSA      EYLCVDHCFPEYDIWTQFVSADYTRAQELDALDNSHPIEVSVGHPSEVDEIFDAISYSKG      180
EmPSA1      EYLAVDHCFPDYDIWTLFVSREYIRALQLDELKSSHPIQVEVNSAREVEEIFDAVSYQKG      180
            ***.******:***** *** :* ** :** *..****:*.*.   **:*****:**.**

Hs_PSA      ASVIRMLHDYIGDKDFKKGMNMYLTKFQQKNAATEDLWESLENASGKPIAAVMNTW    236
EmPSA1      SCVIRMLYNYVGASSFEAGLKSYFKRFKYSNAETQDLWTALEATGVDNLTELMSPW    236
            :.*****::*:* ..*: *:: *:.:*: .** *:*** :** :. . :: :*. *
```

Figure S2C: Sequence features and expression of *EmPSA1*. Depicted are the *E. multilocularis* transcript ID numbers and GenBank accession numbers for *EmPSA1* (above). Upper panel: WISH analysis for *EmPSA1* on *in vitro* cultivated metacestode vesicles. Channel settings were blue (DAPI, nuclei), red (EdU, S-phase GC), and green (WISH+). From left to right: DAPI/EdU, DAPI/WISH+, and merge of DAPI/EdU/WISH+. White arrows mark cells double positive for EdU and WISH. Size bar = 20 µm. Middle panel: Deduced amino acid sequence of EmPOU1. Predicted peptidase_M1 (blue) and ERAP1_C (green) domains are marked. Lower panel: Amino acid sequence comparison between the peptidase_M1 domains of EmPOU1 and human puromycin sensitive aminopeptidase (Hs_PSA; GenBank accession no. P55786). Sites of perfect alignment (*) as well as groups of strong (:) or weak (.) similarity are marked below the alignment.

EmNcoA5 (EmuJ_001142000; OR233049)

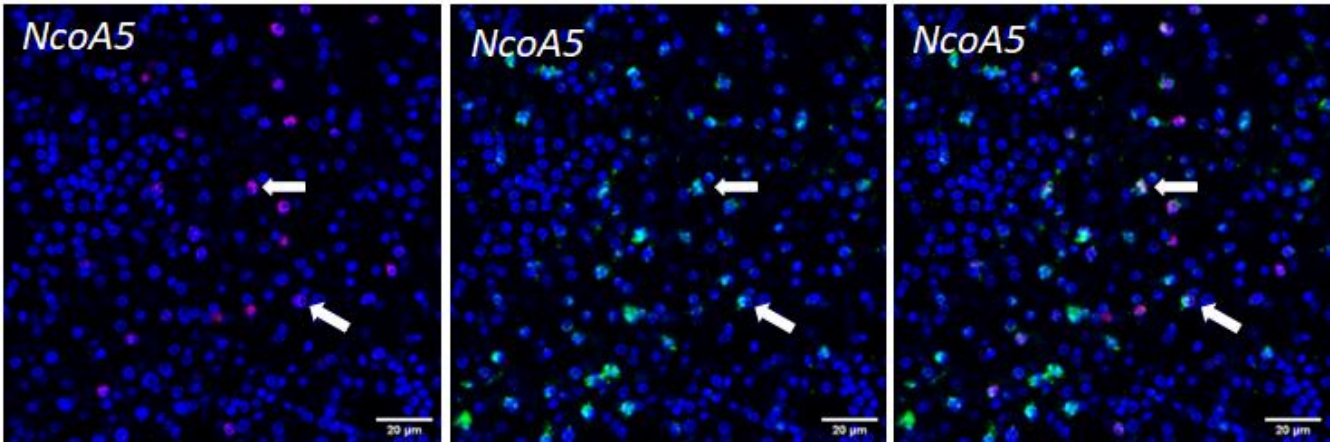


```
MRSRRDLSRSSSKGCLKRVVIEGLKPPFDKDSTIRTWLARYGTVEEIERFYNNLVVKFSSELQAAEAVRC 070
ENGIRSSGSNIKVHLADSETIRQLIQRHSRRKRSLSQQQRRSASSKSESSTRPARKPYLLPPPFPSSRLK 140
TLGSRGLDVKIQAPKRESSAQESREATTSAQLQHQRHSRQLFSTSQSSSEYDVAVLAITHDLVQYAETVQ 210
ALLHRRAIEEGSLLLDHERQKQQQQEVSELHFRSPRVKIMVLMSVDHIAPCMQDLGEEGVLFAILLNVAN 280
MTHNSCTLRILHSSTQQEHRNMPLPDAIDLLLRDFADYLEAEAPSSAGSVAVLKASSPLPLPAPLSPTFQ 350
QHLQQSQQQQSHQQTHHHRRCCRHRHRHTSLSSLSTCTSISSSSLSFRSASSSATSSSNHLQKSRRKSHQ 420
SQQQKQLRLHHRHHHQQQQHLTREEADTSVTALGAETIPAPDDPNFLAPTRHVAVLLRMLADSRILSVGE 490
LDEISAFIAQRRARLTSELTAAESLVHRLSSSPHLCIDTSEALQMNETAFSSASEVIVTPNVGPKEPEFL 560
HREALPVSRYQAIFSDPSNRRRLRAHRGLRGKFRKNGSE                                599
```

```
NCOA5_SCHMD      RSRGIVEEPRSRSQLHQDYPVIPKEIQEPKWNSPPFTDKIGTERPGFDAAIIVMSSELLS 191
EmNcoA5          --------------------TTSAQLQHQRHSRQLFS--TSQSSSEYDVAVLAITHDLVQ 204
                                    .   ::*. : .   *:    . .   :*.*::.:: :*:.

NCOA5_SCHMD      YAETIESRLQRQIA--------------------------LTTHIIVLKEESHANACVED 225
EmNcoA5          YAETVQALLHRRAIEEGSLLLDHERQKQQQQEVSELHFRSPRVKIMVLMSVDHIAPCMQD 264
                 ****::: *:*:                                .:*:** . .*   *::*

NCOA5_SCHMD      LNNKGVLYAFLVNSLNEQHFSCTLRILYG-TPQEHRNMPLNNALTFVARNFERYQKHVRD 284
EmNcoA5          LGEEGVLFAILLNVANMTHNSCTLRILHSSTQQEHRNMPLPDAIDLLLRDFADYLEAEAP 324
                 *.::***:*:*:*  *   * *******:. * ******** :*: :: *:*   * :

NCOA5_SCHMD      L--------KTIAPKPTFDPSRMPPGFIAPDDKIIYYLHLLADSRMLTIEELNEVIDFVQ 336
EmNcoA5          SSAGSVAVLKASSPLPL--PAPLSPTFQQ---------HLQQSQQQQSHQQTHHHRRCCR 373
                          *: :* *   *: : * *            **  ..:  : :: :.     :
```

Figure S2D: Sequence features and expression of *EmNcoA5*. Depicted are the *E. multilocularis* transcript ID numbers and GenBank accession numbers for *EmNcoA5* (above). Upper panel: WISH analysis for *EmNcoA5* on *in vitro* cultivated metacestode vesicles. Channel settings were blue (DAPI, nuclei), red (EdU, S-phase GC), and green (WISH+). From left to right: DAPI/EdU, DAPI/WISH+, and merge of DAPI/EdU/WISH+. White arrows mark cells double positive for EdU and WISH. Size bar = 20 µm. Middle panel: Deduced amino acid sequence of EmNcoA5. A predicted RNA recognition motif (blue) is marked. Lower panel: Amino acid sequence comparison between central regions of EmNcoA5 and Schmidtea mediterranea NcoA5 (NCOA5_SCHMD; GenBank accession no. U6A492). Sites of perfect alignment (*) as well as groups of strong (:) or weak (.) similarity are marked below the alignment.

EmCAF1A (EmuJ_000609400; OR233050)

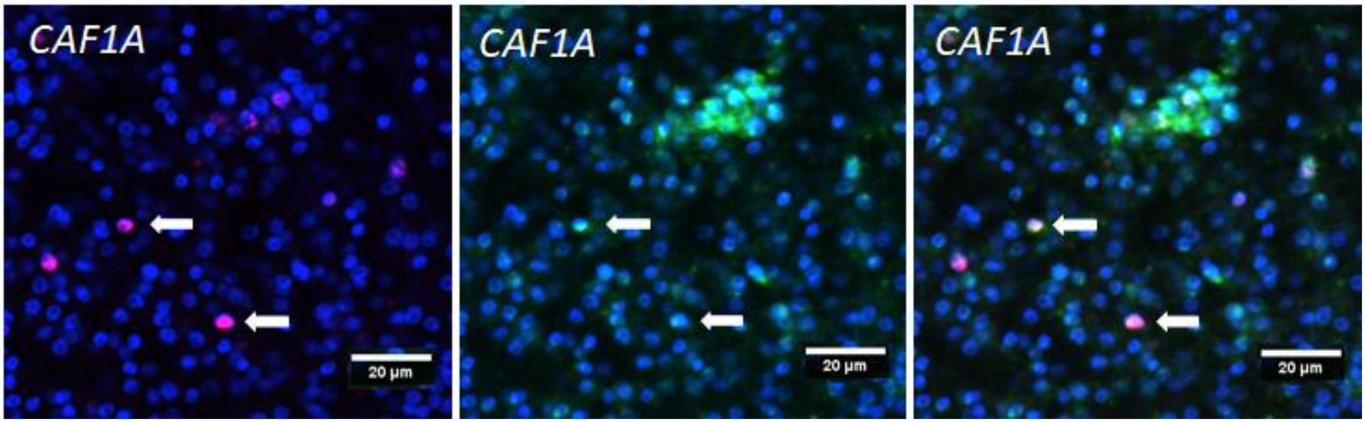


```
MREDGRDTSSEAPCQAETSKKYRNSPRTRQPKSNTPLKRDLKDREKAEKQVERERLRGEKEQRRQERQER 070
REQAEREKEARRLQREEERAKREEERRVREREREDERARKEEVRARKEEGRKAKEEERRKREAERVREEE 140
EKRRKMEKQRALLMGFFVQQGKPENLTTSGAGGGGRSSDYGASTSPFMQFELKRDQRQSPICRVRSEALR 210
HTKWQNVEDLRLSWQSGHSTVDGSRLGLMKFGQPNYLHELRTGRIKPLSFPPTWPIEMPDGGGVSLQNMV 280
ALRFNHGDSGNGGTVWIKAKLLQFFENYRPAFYGTWRRRSYAISPRRPFTKDCYQLDYEVDSDDEWEEEE 350
PGENISQSDGEEEEEVEDDEDEDAKFLVPHGYLSDDEGVHDEDDLEGGVSGIDDSGNGRETAEMKRLRQR 420
LSLAEYEAAHSRGLHKLKPLLLGPVWTRNPLEALLPAEVPPMATGYASDDDKENHIPLSTSDDGKSGGTI 490
WALSSKEEIHFMGTSLCTYRVYLWPGTAPPIVPQTDPTPDIPSGANKPSLTERLKKRFPREAIPYLIRLV 560
HKNALSRVKLQFEFRVFWLKHIWTGEGEAPGCCLSYKEYSQSSEAEATGTPNLPIGQRAADTKPQQQLGN 730
SDSLPLSKTLTMNKISEIAVFEEGLWRVRPEVLKTHRDAVLALPGLPKADESVHAITDPNFVFPSWTYLT 800
DVAVNTIMRRKSLVPSAAASQSPTLPLQPSSSSLPSPLSASRSKVARSTMVKKRATLETFFLAAPPPSAK 870
RKKTSGEVDEVNSSDKGNGEGVGDP                                              895
```

```
Hs_Caf1A      KLLQFCENHRPAYWGTWNKKTALIRARDPWAQDTKLLDYEVDSDEEWEEEEPGESLSHSE   60
EmCaf1A       KLLQFFENYRPAFYGTWRRRSYAISPRRPFTKDCYQLDYEVDSDDEWEEEEPGENISQSD   60
              ***** **:***::***.:::  *  * *:::*   ********:*********.:*:*:

Hs_Caf1A      GDDDDDMGEDEDEDD    75
EmCaf1A       GEEEEEVEDDEDEDA    75
              *:::::: :*****
```

Figure S2E: Sequence features and expression of *EmCAF1A*. Depicted are the *E. multilocularis* transcript ID numbers and GenBank accession numbers for *EmCAF1A* (above). Upper panel: WISH analysis for *EmCAF1A* on *in vitro* cultivated metacestode vesicles. Channel settings were blue (DAPI, nuclei), red (EdU, S-phase GC), and green (WISH+). From left to right: DAPI/EdU, DAPI/WISH+, and merge of DAPI/EdU/WISH+. White arrows mark cells double positive for EdU and WISH. Size bar = 20 µm. Middle panel: Deduced amino acid sequence of EmCAF1A. A predicted CAF1 domain (blue) is marked. Lower panel: Amino acid sequence comparison between CAF1 domains of EmCAF1A and human chromatin assembly factor 1A (Hs_Caf1A; GenBank accession no. Q13111). Sites of perfect alignment (*) as well as groups of strong (:) or weak (.) similarity are marked below the alignment.

EmKIP1 (EmuJ_001180200; OR233051)

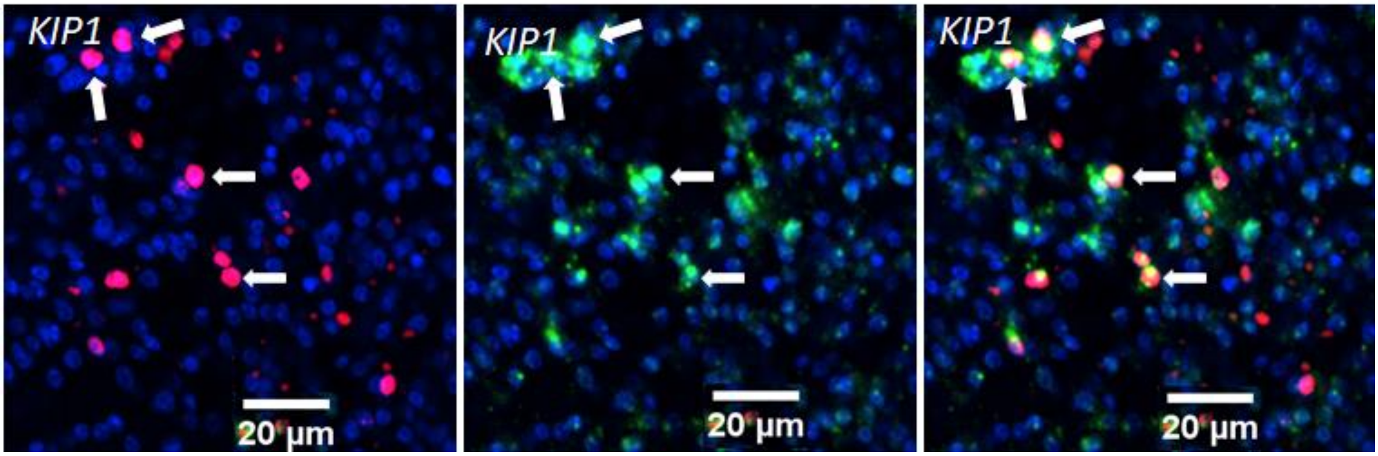


```
MEIVAPTVVEVLEGYNCTIFAYGQTGTGKTFTMTGERSDPLRYTWETDPTAGIVPRALNQIFSTLDMMGCD 070
FSVRVSFLELYNEELFDLLSSGEDCVRLAVYDDANKKGSVLVKGLREVAVHNKEEVYTILERGLLRRQTAS 140
TQLNAQSSRSHTVFTVTVHIKEVCPDDDEVFLKIGKLNLVDLAGSENIGRSGALDKRAREAGSINQSLLTL 210
GRVITCLVDHSPHIPYRESKLTRLLQDSLGGKTKTSIIATIGPGSSSLEETISTLDYAHRAKNIQNRPEIN 280
QKLNKNQLIKGYNEELERLRRDLEAARSKNGIFVDADNYQAMTLQLTQQRVRIDDLESRKETLESDLESAV 350
```

```
Hs_KIF11     TKQIDVYRSVVCPILDEVIMGYNCTIFAYGQTGTGKTFTMEGERSPNEEYTWEEDPLAGI  120
EmKIP1       -------MEIVAPTVVEVLEGYNCTIFAYGQTGTGKTFTMTGERSDPLRYTWETDPTAGI  53
                     .:*.* : **: ******************** ****   .**** ** ***

Hs_KIF11     IPRTLHQIFEKLTDNGTEFSVKVSLLEIYNEELFDLLNPSSDVSERLQMFDDPRNKRGVI  180
EmKIP1       VPRALNQIFSTLDMMGCDFSVRVSFLELYNEELFDLLSSGED-CVRLAVYDDANKKGSVL  112
             :**:*:***..*   * :***:**:**:*********. ..* . ** ::** .:* .*:

Hs_KIF11     IKGLEEITVHNKDEVYQILEKGAAKRTTAATLMNAYSSRSHSVFSVTIHMKETTIDG-EE  239
EmKIP1       VKGLREVAVHNKEEVYTILERGLLRRQTASTQLNAQSSRSHTVFTVTVHIKEVCPDDDEV  172
             :***.*::****:*** ***:*  :* **:* :** *****:**:**:*:**.   *. *

Hs_KIF11     LVKIGKLNLVDLAGSENIGRSGAVDKRAREAGNINQSLLTLGRVITALVERTPHVPYRES  299
EmKIP1       FLKIGKLNLVDLAGSENIGRSGALDKRAREAGSINQSLLTLGRVITCLVDHSPHIPYRES  232
             ::*********************:********.*************.**::**:*****

Hs_KIF11     KLTRILQDSLGGRTRTSIIATISPASLNLEETLSTLEYAHRAKNILNKPEVNQ     352
EmKIP1       KLTRLLQDSLGGKTKTSIIATIGPGSSSLEETISTLDYAHRAKNIQNRPEINQ     285
             ****:*******:*:*******.*.* .****:***:******** *:**:**
```

Figure S2F: Sequence features and expression of *EmKIP1*. Depicted are the *E. multilocularis* transcript ID numbers and GenBank accession numbers for *EmKIP1* (above). Upper panel: WISH analysis for *EmKIP1* on *in vitro* cultivated metacestode vesicles. Channel settings were blue (DAPI, nuclei), red (EdU, S-phase GC), and green (WISH+). From left to right: DAPI/EdU, DAPI/WISH+, and merge of DAPI/EdU/WISH+. White arrows mark cells double positive for EdU and WISH. Size bar = 20 µm. Middle panel: Deduced amino acid sequence of EmKIP1 N-terminal regions. A predicted kinesin motor catalytic domain (blue) is marked. Lower panel: Amino acid sequence comparison between KISc domains of EmKIP1 and human kinesin like protein KIF11 (Hs_KIF11; GenBank accession no. P52732). Sites of perfect alignment (*) as well as groups of strong (:) or weak (.) similarity are marked below the alignment.

EmCIP2Ah (EmuJ_000955000; OR233052)

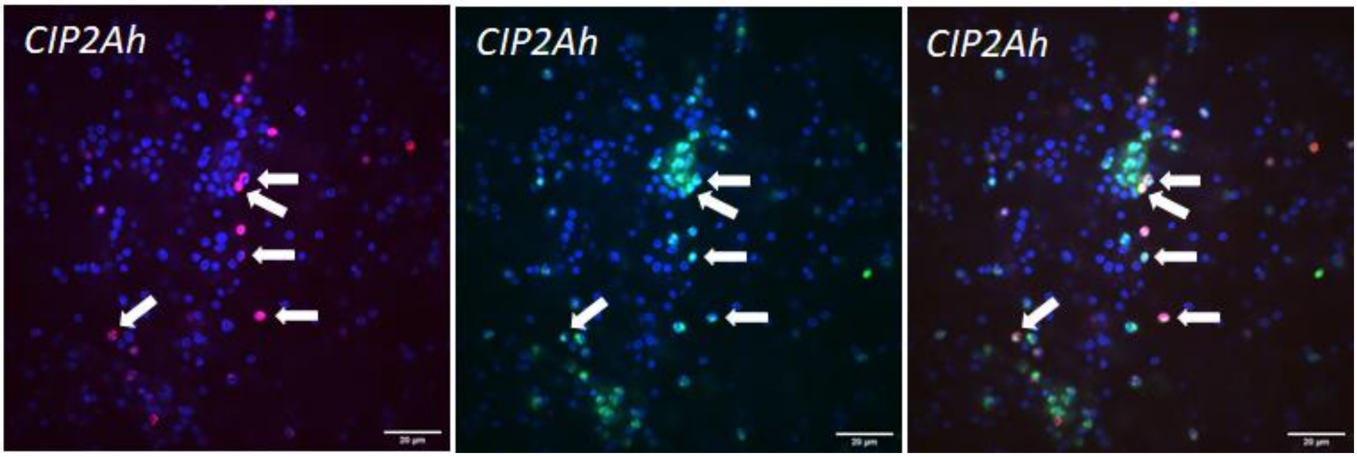


```
CIP2A_Hs      MDSTACLKSLLLTVSQYKAVKSEA----NATQLLRHLEVISG---QKLTRLFTSNQILTS    53
EmCIP2Ah      ----MTVNLILDAFDKYRQNESVDAFVCSMEEIIHKLEKITSLEPDTLDRLN---AFFS-    52
                    :: :* :..:*:  :*      .  :::::** *:.   :.* **     :::

CIP2A_Hs      ECLSCLVELLEDPNISASLILSIIGLLSQLAVDIETRDCLQ-NTYNLNSVLAGVVCRSSH    112
EmCIP2Ah      -FALCSPSGLQLRSIALKVIL-------NYADDMLSRYVLLAKFLILKPILHGIFNWS--    102
                  *  . *:  .*: .:**       : * *: :*  *  :   *: :* *:.  *

CIP2A_Hs      TDSVFLQCIQLLQK---------LTYNVKIFYSGANIDELITFLIDHIQSSEDELKMPCL    163
EmCIP2Ah      EEKIFLEALAAFYKCTFEINYMPLEYEDDIFVCGVRQ--VLSILKAPY---NKEKVEFAL    157
               :.:**:.:  : *         * *: .** .*..   ::::*       :.*    .*

CIP2A_Hs      GLLANLCRHNLSVQTHIKTLSNVKSFYRTLI-TLLAHSSLTVVVFALSILSSLTLNEEVG    222
EmCIP2Ah      GILANISQKHSVIHKVLQNRDDF-DFLRKLLLRIMNSESSTQMETVLS-MTIIFHLWGIK    215
              *:***:.:::  ::. ::. .:. .* *.*:  ::  .* * :  .** :: :     :

CIP2A_Hs      EKLFHARNIHQTFQLIFNILINGDGTLTRKYSVDLLMDLLKNPKIADYLTRYEHFSSCLH    282
EmCIP2Ah      DKFFDSRNAHASLQVLFNIFLSGNASLESLYAGDILIDFCANSTSFSLINSHPKLKHVMI    275
              :*:*.:** * ::*::***::.*:.:*   *: *:*:*:  * .  . :. : ::.  :

CIP2A_Hs      LNKQIETVKKLNESLKEQNEKSIAQLIEKEEQRKEVQNQLVDREHKLANLHQKTKVQEEK    814
EmCIP2Ah      LEAQRDQISR----LESENGRLSSELSSKRKEHESLQSAME-------------SLQNR    815
              *: * : :.:    *:.:* :  ::* .*.::::.:*. :                 :::

CIP2A_Hs      IKTLQKEREDKEETIDILRKELSRTEQIRKELSIKASSLEVQKAQLEGRLEEKESLVKLQ    874
EmCIP2Ah      LTSMREQNRTMDATIDN----------------------------FRKQLDEAKQIIQQK    847
              :.::::::..  : ***                            :. :*:* :.::: :

CIP2A_Hs      QEELNKHSHMIAMIHSLSGGKINPETVNLSI---      905
EmCIP2Ah      DAEIQQYTHMTRIINELTGNRVQPANASNSKNPI      881
              : *:::::**  :*:.*:*.:::* ... *
```

Figure S2F: Sequence features and expression of *EmCIP2Ah*. Depicted are the *E. multilocularis* transcript ID numbers and GenBank accession numbers for *EmCIP2Ah* (above). Upper panel: WISH analysis for *EmCIP2Ah* on *in vitro* cultivated metacestode vesicles. Channel settings were blue (DAPI, nuclei), red (EdU, S-phase GC), and green (WISH+). From left to right: DAPI/EdU, DAPI/WISH+, and merge of DAPI/EdU/WISH+. White arrows mark cells double positive for EdU and WISH. Size bar = 20 µm. Lower panel: Amino acid sequence comparison between regions of EmCIP2Ah and human CIP2A (CIP2A_Hs; GenBank accession no. Q8TCG1). Sites of perfect alignment (*) as well as groups of strong (:) or weak (.) similarity are marked below the alignment.
